# Synthesis, biological evaluation, and computational studies of some novel quinazoline derivatives as anticancer agents

**DOI:** 10.1186/s13065-022-00893-z

**Published:** 2022-11-22

**Authors:** Leila Emami, Soghra Khabnadideh, Zahra Faghih, Farnoosh Farahvasi, Fatemeh Zonobi, Saman Zare Gheshlaghi, Shadi Daili, Ali Ebrahimi, Zeinab Faghih

**Affiliations:** 1 Pharmaceutical Sciences Research Center, Shiraz University of Medical Sciences, Shiraz, Islamic Republic of Iran; 2 Faculty of Pharmacy, Shiraz University of Medical Sciences, Shiraz, Islamic Republic of Iran; 3 Shiraz Institute for Cancer Research, Medical School, Shiraz University of Medical Sciences, Shiraz, Iran; 4 Department of Chemistry, Computational Quantum Chemistry Laboratory, University of Sistan and Baluchestan, Zahedan, Iran; 5 Department of Physical and Environmental Sciences, University of Toronto Scarborough, 1265 Military Trail, Toronto, ON M1C1A4 Canada

**Keywords:** Quinazoline, Synthesis, Anticancer agents, Computational Studies

## Abstract

A series of quinazolinone derivatives (***7a***–***7h***) were synthesized as antiproliferative agents. All compounds, were synthesized through three steps method and structurally evaluated by FTIR, ^1^H-NMR, ^13^CNMR and Mass spectroscopy. Their cytotoxic activities were assessed using MTT protocol against three humans cancerous (MCF-7, A549 and 5637) and normal (MRC-5) cell lines. In addition, molecular docking and simulation studies of the synthesized compounds were performed to assessment their orientation, interaction mode against EGFR as plausible mechanism of quinazoline compounds as anticancer agents. The synthesized compounds mostly showed moderate activity against the three studied cell lines. They also indicated an appropriate selectivity against tumorigenic and non-tumorigenic cell line. The molecular docking results also confirmed biological activity. Most of the compounds fulfilled Lipinski rule. Collectively, these compounds with further modification can be considered as potent antiproliferative agents.

**Supplementary Information:**

The online version contains supplementary material available at 10.1186/s13065-022-00893-z.

## Introduction

**Fig. 1:**
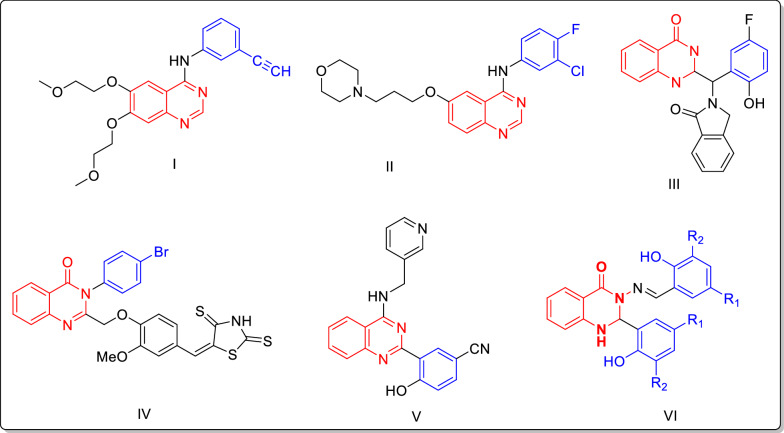
Several marketed and reported anticancer agents with quinazoline scaffold

Cancer is a complicated disease due to uncontrolled growth of cells without differentiation, and an increase in abnormal cells leading to tumor formation [1, 2]. In 2020, one out of every 6 deaths in the world was due to cancer and approximately 10 million people died from cancer that year. Breast, lung, colon, rectum and prostate cancers are the most common cancers worldwide [3]. Chemotherapy, surgery, hormone therapy and radiotherapy are the main cancer treatments based on the stage and type of cancer [4]. Nevertheless, multidrug resistance (MDR) and healthy cell damage during cancer treatment are among the main disadvantages of these treatments [5]. Therefore, there is an urgent need to find novel and selective compounds as antiproliferative agents. Quinazoline scaffolds have been shown to have various biological and pharmacological effects including anti-cancer [6–8], anti-diabetes [9], antifungal [10], antibacterial [11, 12], antihypertensive [13] and anti-tuberculosis activity [14]. In addition, there are various quinazoline scaffold based compounds in the market such as erlotinib, gefitinib (structures I and II) and structures III–VI, with high cytotoxic activity toward different cancerous cell lines (Fig. 1) [15–18].

**Fig. 2:**
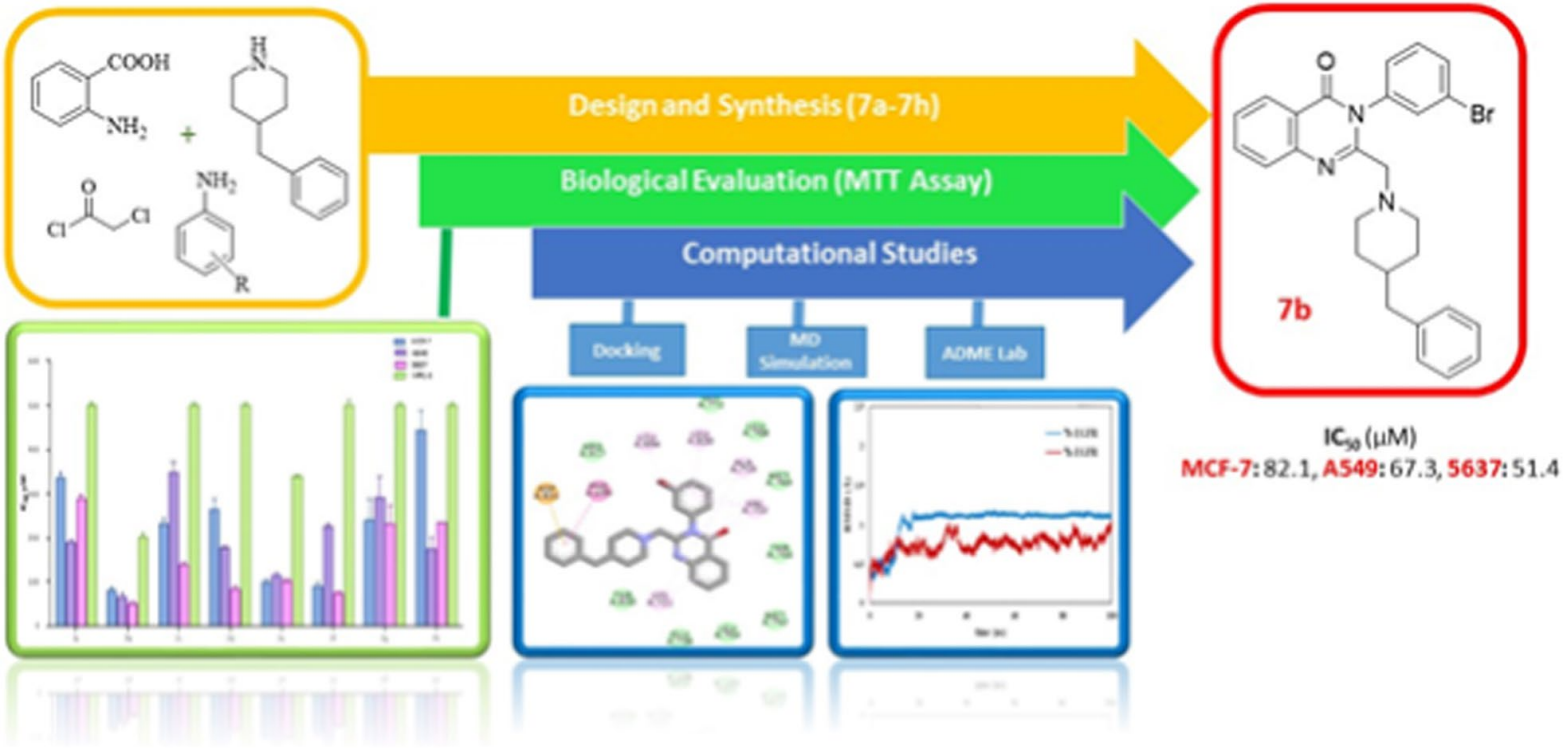
The summery schematic of the study

Inhibition of epidermal growth factor receptor (EGFR) enzyme is considered to be one of the main mechanisms of quinazolinone analogs as anticancer agents [19]. EGFR is a receptor with tyrosine kinase (TK) activity with significant designation in the cell proliferation, differentiation, metastasis, and survival cycle [20, 21]. Over activation of EGFR has been reported in some cancerous tissues such as lung, brain, ovarian, colon, breast and prostate tumors [22]. Thus, targeting EGFR can be considered as a rational and worthy approach in cancer therapy. Erlotinib and gefitinib are the most potent and selective EGFR inhibitors with quinazoline scaffold in their structures. Considering the importance of quinazoline and quinazolinone base structures in cancer treatment, in this study, we synthesized some novel quinazolinone-benzyl piperidine derivatives (***7a***–***7h***) to obtain more effective anticancer agents. All the synthesized derivatives were elucidated with ^1^HNMR, ^13^CNMR, FT-IR and Mass spectroscopy. All compounds were then evaluated against three humans cancerous (MCF-7, A549 and 5367) as well as one normal (MRC-5) cell lines. In addition, these quinazolinone-benzyl piperidine derivatives were applied to a molecular docking simulation to acquire their binding conformations and structural specificities toward EGFR kinase as plausible targets in cancer treatment. Finally, In silico physico-chemical properties were also performed to represent drug-likeness of synthesized compounds. The summery schematic (graphical abstract) of our study was shown in Fig. 2.

## Results and discussion

### Chemistry

The synthetic route of the compounds (***7a***–***7h***) via three different steps is shown in Fig. 10. The first and second steps were performed as previously described [23]. The final target compounds (***7a***–***7h***) were obtained by reacting 4-benzyl piperidine with intermediates (***5a***–***5h***) through nucleophilic substitution in the presence of basic catalyst (DIPEA) and acetonitrile as solvent in appropriate yields (50–95%). The chemical structures of all compounds were confirmed by IR, ^1^H-NMR, ^13^C-NMR, and mass spectroscopies. The important feature of the ^1^H NMR spectrum of these compounds is a double peak at 2.54–3.31 ppm belonging to two protons of CH_2_ placed between quinazolinone and 4-benzyl piperidine ring, except for compounds ***7a***, ***7b*** and ***7d***. The peaks appearing as two peaks of doublet may be due to nonequivalent protons in these compounds. Only in compound ***7d***, the peaks of CH_2_ between phenyl and piperidine ring appeared as a singlet, while for other compounds it appeared as a doublet, triplet or multiplet. Regarding the ^13^C NMR spectrum, the significant singlet peak is related to the carbonyl group in quinazoline ring, which was displayed in the range of 161.1–161.6 ppm.

### Cytotoxic activity

The antiproliferative activities of all the synthesized compounds were assessed by MTT method on three cancerous (MCF-7, A549 and 5367) as well as one normal cell line (MRC-5). According to the results, among mono-substituted compounds (***7a***, ***7b***, ***7d***–***7g***), ***7b*** with bromine substitution at the meta position of phenyl ring had greatest effect compared to other studied compounds with IC_50_ values of 82.1 µΜ, 67.3 µΜ and 51.4 µΜ against cancerous cell lines MCF-7, A549 and 5367 respectively. This effect could be related to the size and electronegativity of bromine. In addition, compound ***7e*** containing chlorine atom at para position of phenyl ring showed IC_50_ values of 90.2 and 103.04 µΜ for MCF-7 and 5637 cell lines, respectively. Moving of the chlorine atom from para to meta position led to decreasing activity in ***7a*** analogue. In case of disubstituted compounds (***7c*** and ***7h***), compound ***7c*** containing dimethoxy substitutions is more effective than ***7h*** with methyl and Cl substitutions in MCF-7 and 5637 cancerous cell lines. Overall, the mono-substituted groups showed better effects than the disubstituted compounds (Table 2). All compounds generally represented lesser anti-proliferative effects on the normal cell line (MRC-5) compared to other carcinoma cell lines, which illustrates appropriate selectivity between non-tumorigenic and tumorigenic cell lines [24].

### Docking studies

**Fig. 3:**
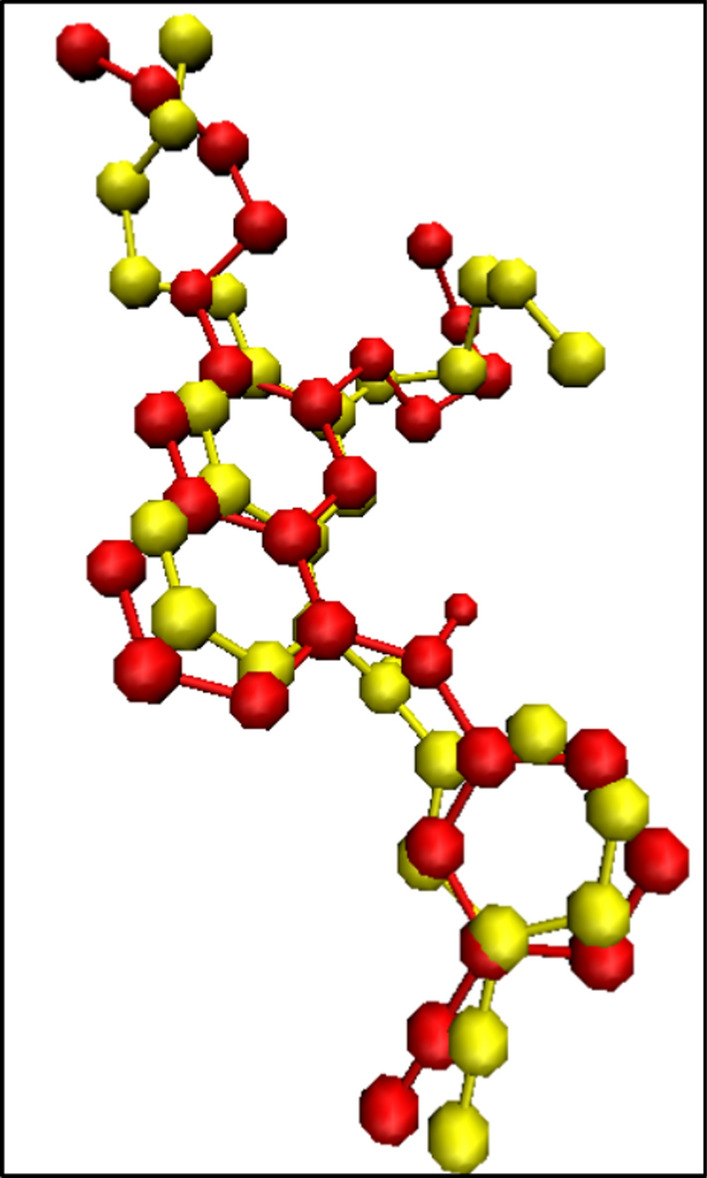
Two conformations of co-crystal ligand (erlotinib) (AQ4) in the EGFR active site: The yellow and red color showed the crystal orientation and redocked conformation, respectively

As mentioned previously, EGFR is the most plausible target for the compounds with quinazoline backbone as anticancer agents. Therefore, to evaluate and understand the pattern of interaction and binding mode of the synthesized compounds in the active site of EGFR, molecular modeling was performed. The docking binding energies and interaction of all synthesized compounds were shown in Table 3. As shown in Table 3, compound ***7b*** and ***7e*** with high anti-proliferative activity, showed stronger energies in binding to the active sites compared to the others. Redocking of [6,7-Bis(2-Methoxy-Ethoxy) Quinazoline-4-Yl]- (3-Ethynylphenyl) Amine (erlotinib) as co-crystal ligand, was performed to evaluate the docking accuracy. The RMSD was achieved 1.07 Å compared to its coordination in the crystal structure (Fig. 3). The docking score of erlotinib was obtained − 10.1 (kcal/mol).

**Fig. 4:**
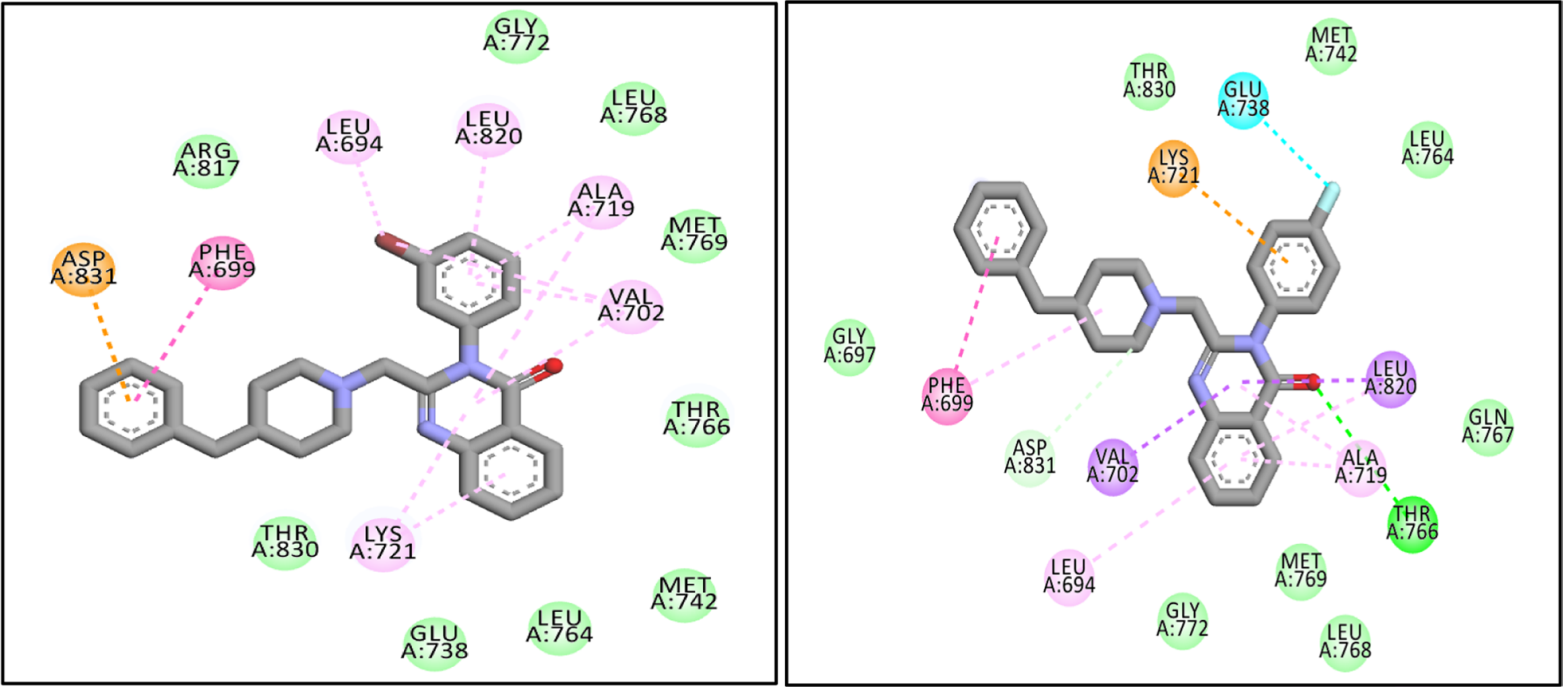
2D interactions of ***7b*** (left) and ***7e*** (right) with the residues in the binding site of receptor. (Vander waals: green, dark pink: pi-pi, light pink: pi-alkyl, purple: pi-sigma, orange: pi-cation, blue: halogen bond)

The interactions of two synthesized quinazolinone-benzyl piperidine (***7b*** and ***7e***), as the most active compounds, with EGFR were investigated. As shown in Fig. 4, compound ***7b*** showed pi-anion interaction between 4-benzyl piperidine and Asp 831 and also, pi-alkyl interactions were seen between quinazoline ring and 3-Br benzyl and Leu 694, Leu 820, Ala 719, Val 702, Lys 721. There are some hydrophobic interactions with Arg 817, Thr 830, Glu 738, Leu 764, Met 742, Thr 766, Met 769, Leu 768, Gly 772.

The docking scores and detailed interactions of all designed compounds (***7a–7h***) are shown in Table 3.

The other potent molecule (***7e***), interacted through six types of interactions including; hydrogen bonding, halogen bonding, pi-pi interaction, and hydrophobic interaction. The carbonyl group of the quinazoline moiety was involved in hydrogen bond interaction with Thr 766. Furthermore, the quinazolinone and phenyl rings interacted with Leu 820 and Val 702, respectively, via pi-sigma interactions. The other interactions were pi-pi and pi-alkyl interactions between quinazolinone moiety and 4-benzyl piperidine with Ala 719, Leu 694 and Phe 699. Some hydrophobic interactions with Gly 697, Gly 772, Met 769, Leu 768, Gln 767, Leu 764, Met 742, Thr 830 were also observed in Fig. 4.

**Fig. 5:**
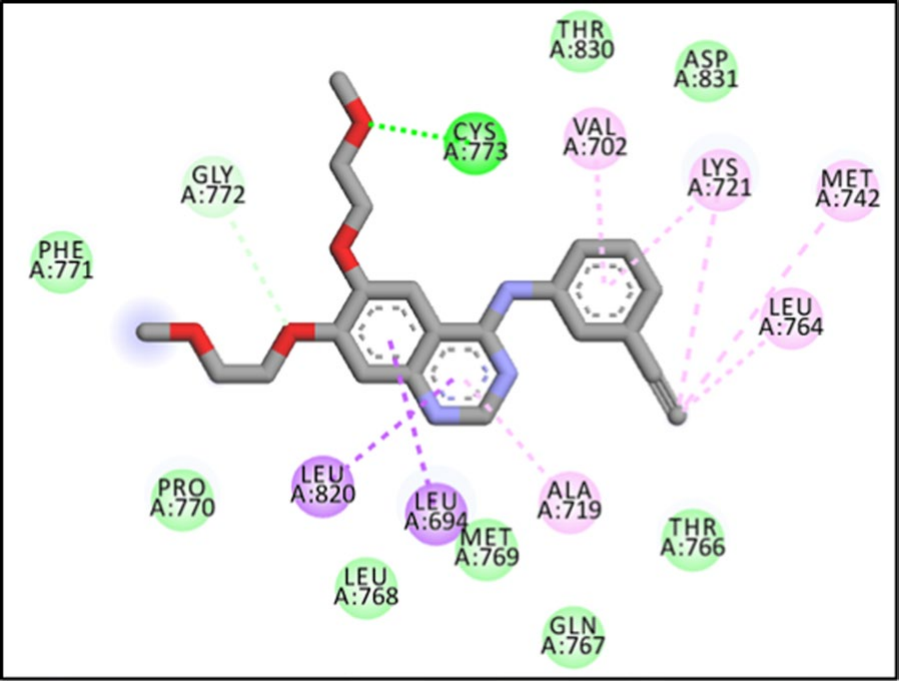
2D interaction of erlotinib with the residues in the binding site of EGFR target. (Vander waals: green, dark pink: pi-pi, light pink: pi-alkyl, purple: pi-sigma, orange: pi-cation, blue: halogen bond)

According to the proposed binding mode of erlotinib (Fig. 5), the quinazoline moiety involved in pi–pi interaction with Leu 694, Leu 820 and Ala 719 and also, the 2-methoxy-ethoxy chain interacted via hydrogen bond with Cys 773. 3-ethynyl phenyl ring showed pi-alkyl interaction with Val 702, Lys 721, Met 742 and Leu 764. Besides, there were some hydrophobic interactions with Phe 771, Pro 770, Leu 768, Met 769, GLN 767, Thr 766, Thr 830and Asp 831.

The results of docking study indicated that, the most active compounds and erlotinib showed the same interactions with key amino acids in the active site of enzyme including Thr 766, Met 769, Ala 719, Lys 721 and Gly 772. However, the less docking score of these compounds compared to erlotinib, probably, can be related to the absence of polar groups attached to the quinazoline ring which increase the inhibitory effect due to the formation of hydrogen bonds in the active site of EGFR enzyme. Altogether, Val 702, Phe 699, Arg 817, Asp 831, Glu 738, Leu 694, Leu 820 and Lys 721 were the most important amino acids for generating interactions with all ligands in the active site of EGFR target.

### *Insilico* physicochemical parameters (ADME) prediction

ADME properties of the all synthesized compounds are represented in Table 4. It can be observed that all compounds follow Lipinski’s rule of five except for compound ***7h***. Molecular weight (MW) of all compounds are in the accepted range (427–502). All of the compounds also showed reasonable lipophilicity (log P values) for penetration through biological membranes. Furthermore, the hydrogen bond properties (donors or acceptors), total polar surface area (TPSA), and rotatable bond number of all investigated compounds is within the acceptable limit. As a results, it can be proposed that these compounds could potentially be administered orally.

### Molecular dynamic simulation

**Fig. 6:**
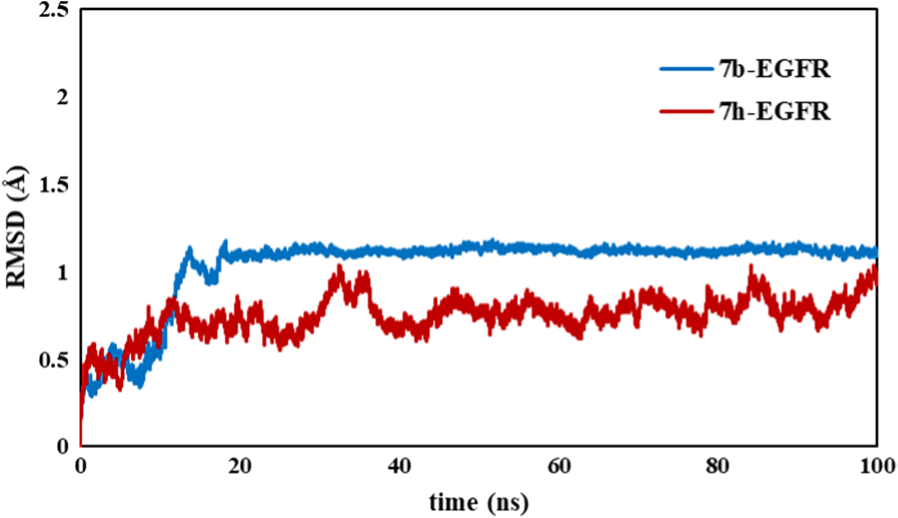
The RMSD plots for protein- ligand complexes during the simulation time

**Fig. 7:**
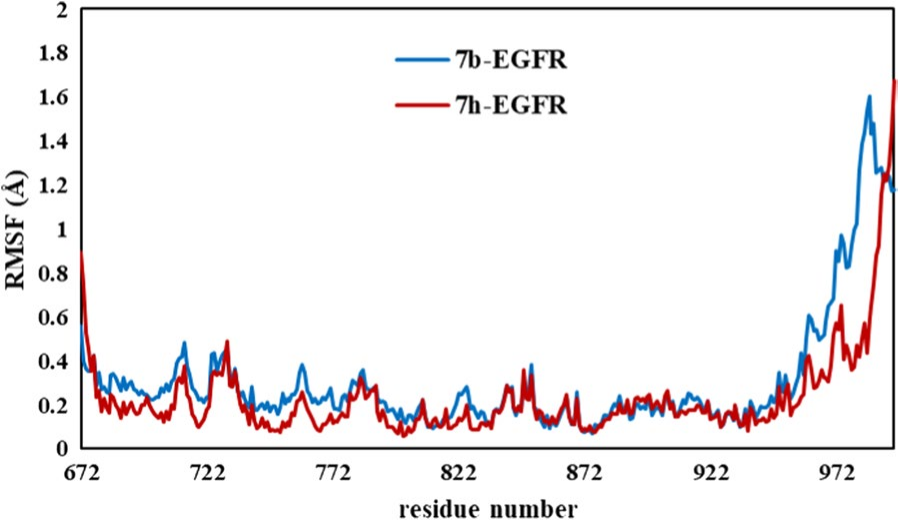
RMSF distribution of residues during the simulation time

**Fig. 8:**
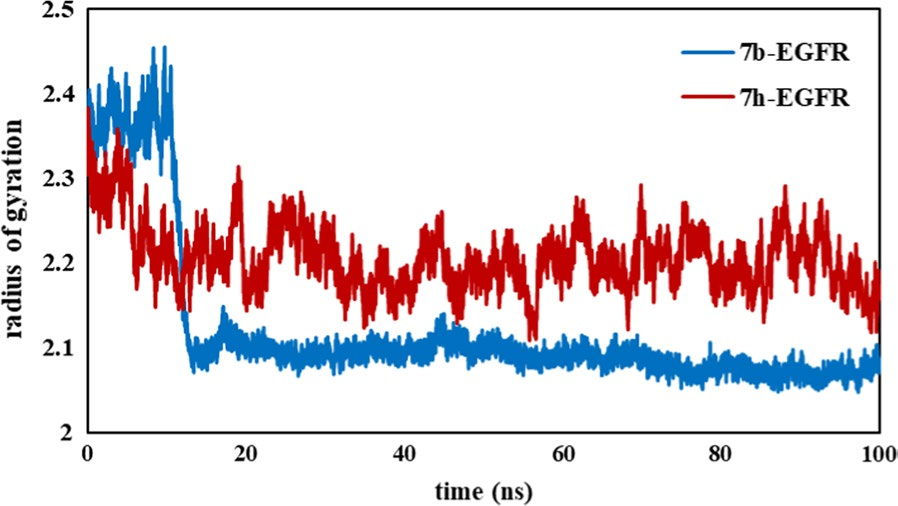
Compactness changes of ligand-EGFR complexes analyzed by Rg parameters

**Fig. 9:**
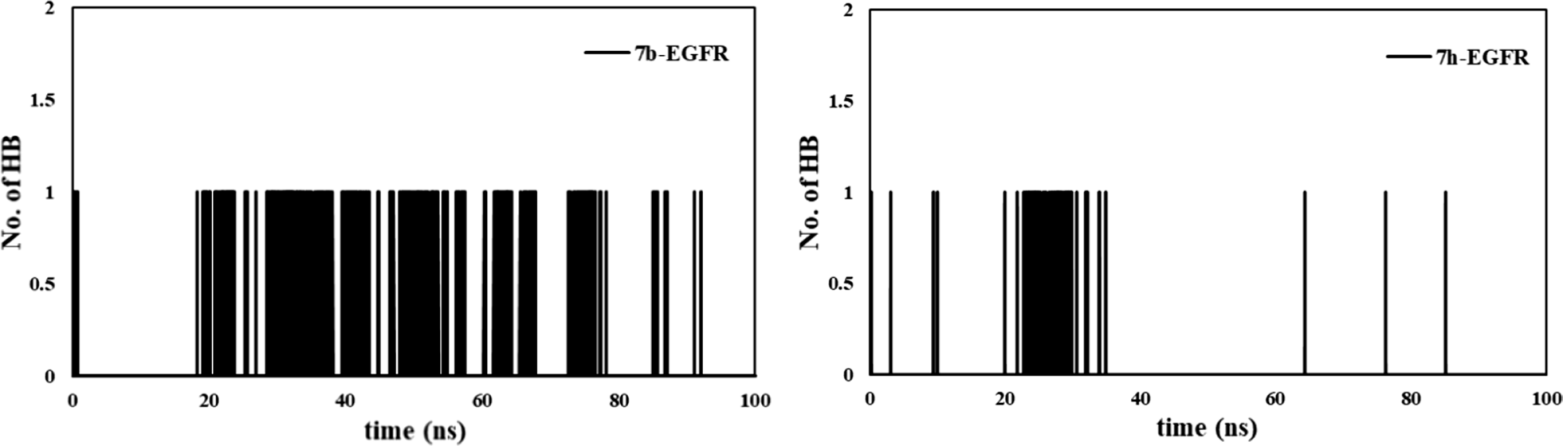
Number of hydrogen bonds between the EGFR and the selected ligands during the simulation time

MD simulation is a powerful method to predict the structural refinements as well as behavior of ligand and receptor in biological systems [25, 26]. To investigate the stability and intramolecular interactions of our compounds (***7b*** and ***7h***) in the active site of EGFR enzyme with respect to time, molecular dynamics simulation was performed [27]. The RMSD, RMSF, number of hydrogen bonds, and radius of gyration (Rg) graphs of ***7b*** and ***7h*** with EGFR receptor are presented in Figs. 6, 7, 8 and 9, respectively.

In general, RMSD analyses shows the stability and equilibrium of the complex during the simulation time. As depicted in Fig. 6, the EGFR complex with ***7b*** has reached equilibrium after 20 ns and remains stable with low fluctuation during the MD simulation time. The average RMSD of ***7b*** in complex with 1M17 was 1A. The complex reached a plateau form and remained stable after 20 ns. The condition for ***7h***-complex is different and the structural changes were continued until the end of the simulation time. This indicates that ***7h*** may show different performances to the ***7b*** ligand. This finding also revealed that ***7b*** fits more appropriately in the active site of EGFR enzyme.

RMSF (root means square fluctuation) results revealed the region of the fluctuated protein during the simulation time. In fact, the amount of structural movement and flexibility of amino acids was achieved with RMSF analysis [28]. The RMSF of the backbone residue of EGFR was performed to calculate the fluctuation of amino acid residues. The lowest RMSF values represented the flexibility, compactness of the protein, and stability of the complex. In fact, RMSF was applied to evaluate the structural movement and flexibility of EGFR in binding to ***7b*** and ***7h***. As shown in Fig. 7, the same distribution of RMSFs for both complex ***7b*** and ***7h*** was observed. It can be concluded that the region around 672–722 and 872–972 which indicates lower fluctuation are active sites of EGFR. Interaction and binding conformation of the ligands ***7b*** and ***7h*** in the IM17 binding site was confirmed before and after MD simulation. The key amino acids Met 769, Leu 768, Leu 694, Gly 772, Asp 776, Gly 695, Phe 699, Asp 831, Cys 773, Val 702, Glu 738, Thr 766, Arg 817, Thr 830, Leu 764, Met 742, Leu 768, Asn 819, Lys 851, Cys 721, Ile 735, Gly 833, Leu 834, Leu 820, Ala 719, Lys 721, Gly 697, Leu 775 in the active site of EGFR enzyme with relatively low RMSF values.

The radius of gyration (Rg) of a protein is reflected in the compactness of protein during the simulation time [29]. The plot of the EGFR radius of gyration during the MD simulations time is shown in Fig. 8. The low values of Rg reflect the compactness and stability of protein. As shown in Fig. 8, after 20 ns, complex ***7b*** was compacted and maintained constant interaction with ***7b***. In the case of ***7h*** complex, both the amount and variation of compactness were increased during the simulation time. It is concluded that ***7b*** is a more suitable inhibitor of EGFR in contrast to ***7h***.

The number of intermolecular hydrogen bonds in the ligand-EGFR complex contribute to the stability of the complex. The analysis of H-bond interactions was performed and shown in Fig. 9. The ***7b-***EGFR complex showed more interactions with active site residues over the 100 ns simulation compared to ***7h*** ligand.

## Experimental

All reagents and solvents were purchased from Merck and Sigma chemical companies without any purification. Compound purification was done by flash chromatography (silica gel 60) or recrystallization. The ^1^H NMR, ^13^C NMR spectra and FT-IR were recorded on a BRUKER DRX-500 AVANCE 400 MHz and a VERTEX70 spectrometer (Bruker, Germany), respectively. Mass spectra were recorded on Agilent Mass instrument using (M++1) mode. Melting point was achieved on Electrothermal 9200 instrument. The chemical structures of all synthesized compounds were shown in Table 1.

### Synthesis and characterization

#### General procedure for the synthesis of substituted 2-((4-benzylpiperidin-1-yl) methyl)-3-phenylquinazolin-4(3H)-one

**Fig. 10:**
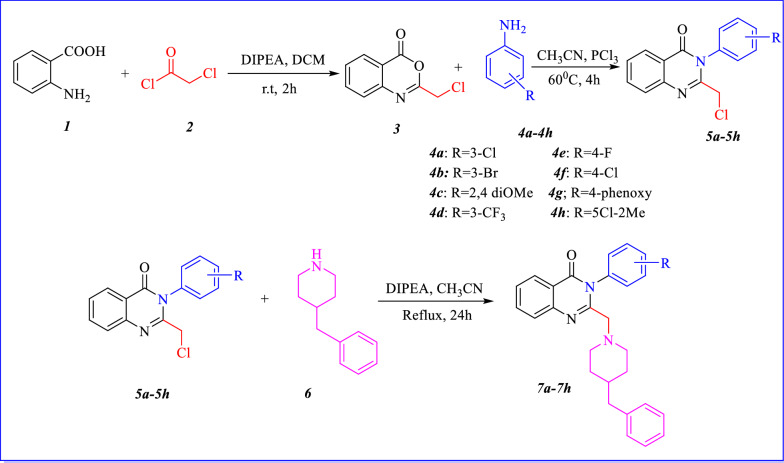
Synthesis path of designed quinazoline (***7a–7h***)

The benzo oxazine [3] was prepared by mixing 1 mmol of anthranilic acid with chloroacetyl chloride in dichloromethane (10 mL) and diisopropylethylamine (DIPEA) (1.5 mmol). After that intermediate 3 was reacted with eight different substituted of aniline (***4a***–***4h***) to obtain compounds (***5a***–***5h***) [23]. Finally, 4-benzyl piperidine (1 mmol) was added to various derivatives of compounds (***5a***–***5h***) (1 mmol) in the presence of diisopropylethylamine (DIPEA) (2 mmol) in acetonitrile as solvent. The mixture was refluxed for 24 h and the reaction was follow up with TLC. The reaction was then washed with water and extracted with suitable amount of ethyl acetate. The final products (***7a***–***7h***) were purified by flash chromatography (Fig. 10).

##### Spectra data

***2-((4-Benzylpiperidin-1-yl) methyl)-3-(3-chlorophenyl) quinazolin-4(3H)-one (7a)***

IR (KBr) v (cm^−1^): 3024–3072 (C-H, aromatic), 2795–2920 (C–H, aliphatic), 1683(C=O, Stretch), 1597(C=C, aromatic), 1468 (C=N, Stretch), 1335 (C-N, Stretch). ^1^H-NMR (400 MHz, DMSO) δ(ppm) = 8.24 (d, 1H, *J* = 10 Hz, H-5-quinazolinone), 7.98 (dd, 1H, *J* = 10 Hz, *J* = 1.5 Hz, H-7-quinazolinone), 7.84 (d, 1H, *J* = 9.5 Hz, H-8-quinazolinone), 7.72–7.73 (m, 1H, H-6-quinazolinone), 7.69–7.70 (m, 1H, aromatic), 7.66–7.67 (m, 1H, aromatic), 7.62–7.64 (m, 1H, aromatic), 7.55 (d, 1H, *J* = 15 Hz, aromatic), 7.37 (t, 2H, *J* = 9.5 Hz, benzyl), 7.27 (t, 1H, *J* = 9 Hz, benzyl), 7.22 (d, 2H, *J* = 8.5 Hz, benzyl), 3.35 (d, 1H, *J* = 17, N–CH_2_–C=N), 3.25 (d, 1H, *J* = 16.5, N–CH_2_–C=N), 2.56–2.62 (m, 3H, N-CH_2_), 2.26–2.30 (m, 1H, CH_2_–CH–CH_2_), 1.91–1.98 (m, 2H, Ph-CH_2_), 1.44–1.50 (m, 3H, N–CH2, N–CH_2_–CH_2_), 1.02–1.17 (m, 2H, N–CH_2_–CH_2_). ^13^C-NMR (100 MHz, DMSO) δ (ppm) = 161.5, 153.4, 146.6, 140.2, 138.4, 138.7, 132.4, 130.1, 129.9, 128.9, 128.4, 128.1, 127.6, 127.2, 127.2, 126.3, 125.7, 120.8, 61.5, 52.9, 52.3, 42.4, 36.9, 31.6, 31.5. MS m/z (%):444.0 [M^+^] (0.82), 269.0 (55.4), 234.0 (14.96), 174.0 (100), 119.0 (9.2), 91.0 (17.8). Elem. anal. calcd. For C_27_H_26_ClN_3_O (443.1); C, 73.04; H, 5.90; N, 9.46. Found: C, 73.05; H, 5.88; N, 9.37.

***2-((4-Benzylpiperidin-1-yl) methyl)-3-(3-bromophenyl) quinazolin-4(3H)-one (7b)***

IR (KBr) v (cm^−1^): 3025–3071 (C–H, aromatic), 2795–2922 (C–H, aliphatic), 1684 (C=O Stretch), 1580–1600 (C=C, aromatic), 1466 (C=N, Stretch), 1335 (C-N, Stretch). ^1^H-NMR (500 MHz, DMSO) δ(ppm) = 8.23 (d, 1H, *J* = 10 Hz, H-5-quinazolinone), 7.94–7.98 (m, 1H, H-7-quinazolinone), 7.82 (dd, *J* = 15 Hz, *J* = 5 Hz, 2H, aromatic), 7.77–7.80 (m, 1H, H-8-quinazolinone), 7.64–7.707 (m, 1H, H-6-quinazolinone), 7.53–7.59 (m, 2H, aromatic), 7.34 (t, 2H, *J* = 9 Hz, benzyl), 7.23–7.27 (m, 1H, benzyl), 7.20 (d, 2H, *J* = 8.5 Hz, benzyl), 3.35 (d, 1H, *J* = 17, N–CH_2_–C=N), 3.22 (d, 1H, *J* = 17, N–CH_2_–C = N), 2.60–2.61 (m, 1H, N–CH_2_), 2.51–2.57 (m, 2H, N-CH_2_), 2.21–2.24 (m, 1H, CH_2_–CH–CH_2_), 1.89–1.96 (m, 2H, Ph-CH_2_), 1.41–1.49 (m, 3H, N–CH2, N-CH_2_-CH_2_), 1.03–1.15 (m, 2H, N–CH_2_–CH_2_). ^13^C-NMR (125 MHz, DMSO) δ (ppm) = 161.5, 153.4, 146.6, 140.2, 138.5, 134.7, 132.9, 131.2, 130.2, 128.7, 128.1, 127.8, 127.2, 127.1, 126.3, 125.7, 120.8, 120.6, 61.5, 53.0, 52.1, 42.5, 36.9, 31.6, 31.5. MS m/z (%):0.488.0 [M^+^] (0.54), 315.0 (29.9), 234.0 (21.0), 174.0 (100), 119.0 (7.6), 91.0 (16.6). Elem. anal. calcd. For C_27_H_26_BrN_3_O (488.4); C, 66.40; H, 5.37; N, 8.60. Found: C, 66.37; H, 5.32; N, 8.52.

***2-((4-benzylpiperidin-1-yl) methyl)-3-(2, 4-dimethoxyphenyl) quinazolin-4(3H)-one (7c)***

IR (KBr) v (cm^−1^):3072(C–H, aromatic), 2772–2930(C–H, aliphatic), 1686(C=O, Stretch), 1606(C=C, aromatic), 1462–1508 (C=N, Stretch), 1313 (C–N, Stretch), 1050–1150 (COC, Stretch). ^1^H-NMR (500 MHz, DMSO) δ(ppm) = 8.23 (d, 1H, *J* = 8.5 Hz, H-5-quinazolinone), 7.93–7.97 (m, 1H, H-7-quinazolinone), 7.90 (d, 1H, *J* = 10 Hz, H-8-quinazolinone), 7.65 (t, 1H, *J* = 9.5 Hz, H-6-quinazolinone), 7.34–7.39 (m, 3H, aromatic), 7.27 (d, 1H, *J* = 9 Hz, benzyl), 7.22 (d, 2H, *J* = 9 Hz, benzyl), 6.79–6.80 (m, 1H, benzyl), 6.71–6.74 (m, 1H, benzyl), 3.94 (s, 3H, OCH_3_-phenyl), 3.78 (s, 3H, OCH_3_-phenyl), 3.34 (d, 1H, *J* = 16.5, N–CH_2_), 3.13 (d, 1H, *J* = 16.5, CH_2_–CH–CH_2_), 2.54 (d, 2H, *J* = 10 Hz, N–CH_2_–C=N), 2.44–2.47 (m, 2H, N–CH_2_), 1.92–1.97 (m, 2H, Ph-CH_2_), 1.45 (s, 3H, N–CH_2_, N–CH_2_–CH_2_), 1.04–1.15 (m, 2H, N–CH_2_–CH_2_). ^13^C-NMR (125 MHz, DMSO) δ (ppm) = 161.1, 160.7, 155.4, 154.8, 146.8, 140.2, 134.6, 130.2, 128.9, 127.1, 126.9, 126.3, 125.7, 120.7, 118.3, 104.4, 98.4, 64.42, 55.5, 53.1, 52.9, 42.3, 36.8, 31.5, 31.3. MS m/z (%): 470.0 $[M+]$ (2.5), 296.0 (32.4), 281.0 (16.4), 265.0 (100), 250.0 (5.9), 234.0 (6.9), 174.0 (96.3), 91.0 (17.1). Elem. anal. calcd. For C_29_H_31_N_3_O_3_ (470.0); C, 74.18; H, 6.65; N, 8.95. Found: C, 74.12; H, 6.61; N, 8.91.

***2-((4-benzylpiperidin-1-yl) methyl)-3-(3-(trifluoromethyl phenyl) quinazolin-4(3H)-one (7d)***

IR (KBr) v (cm^−1^): 3028–3083 (C–H, aromatic), 2764–2929(C–H, aliphatic), 1677 (C=O, Stretch), 1606 (C=C aromatic), 1447 (C=N, Stretch), 1098(C-F, aliphatic). ^1^H-NMR (500 MHz, DMSO) δ(ppm) = 8.13(d, 1H, *J* = 7 Hz, Quinazoline),7.73–7.89(m,6H, Quinazoline and Phenyl),7.57(t, 1H, *J* = 7 Hz, Phenyl), 7.23 (d, 2H, *J* = 7 Hz, Benzyl), 7.14–7.15(m, 1H, Benzyl), 7.07(d,2H, *J* = 7 Hz, Benzyl), 3.29(d, 1H, *J* = 10.8, N–CH_2_–C=N), 3.13(d, 1H, *J* = 10.8, N–CH_2_–C=N),2.35–2.5(m, 2H, N–CH_2_), 2.11–2.13 (m, 1H, CH_2_–CH–CH_2_), 1.8 (s, 2H, Ph-CH_2_), 1.22–1.36 (m,4H, N–CH_2_–CH_2_, N–CH_2_), 0.79–0.93 (m, 2H, N–CH_2_–CH_2_). ^13^C–NMR (125 MHz, DMSO) δ (ppm) = 161.1, 152.7, 146.1, 139.7, 137.4, 134.1, 132.2, 129.1,128.7, 127.6, 126.7, 126.6, 126.4, 125.8, 125.1, 124.6, 124.5, 120.3, 61.2, 52.4, 51.6, 41.8, 36.2, 30.8, 30.7. MS m/z (%): 477.2 (0.4), 303.1 (25.1), 174.3 (80.8), 145.1 (22.4), 119.1 (25.4), 91.2 (29.4), 57.2 (100), 55.2 (82.1). Elem. anal. calcd. For C_28_H_26_F_3_N_3_O (477.5); C, 70.43; H, 5.49; N, 8.80. Found: C, 70.23; H, 5.42; N, 8.71.

***2-((4-benzylpiperidin-1-yl) methyl)-3-(4-fluorophenyl) quinazolin-4(3H)-one (7e)***

IR (KBr) v (cm^−1^): 3025 (C–H, aromatic), 2774–2937 (C–H, aliphatic), 1693 (C=O, Stretch), 1605–1506 (C=C aromatic), 1464 (C=N, Stretch). ^1^H-NMR (400 MHz, DMSO) δ(ppm) = 8.13 (dd, 1H, *J* = 8 Hz, 1.2 Hz, Quinazoline), 7.86(t, 1H, *J* = 7.6 Hz, Quinazoline), 7.73 (d, 1H, *J* = 7.6 Hz, Quinazoline), 7.57 (t, 1H, *J* = 8 Hz, Quinazoline), 7.47–7.51 (m, 2H, Phenyl), 7.34 (t, 2H, *J* = 8.8 Hz,Phenyl), 7.26 (t, 2H, *J* = 7.2 Hz, Benzyl), 7.17 (d, 1H, *J* = 7.2 Hz, Benzyl), 7.12 (d, 2H, *J* = 7.2 Hz, Benzyl), 3.16 (s, 2H, N–CH_2_–C=N), 2.43 (d, 2H, *J* = 6.4 Hz,N–CH_2_), 2.34(d, 2H, *J* = 10.8 Hz, Ph-CH_2_), 1.84(t, 2H, *J* = 10.8, N-CH_2_), 1.37 (d, 3H, *J* = 10.8,N–CH_2_–CH_2,_CH_2_–CH–CH_2_), 0.96–1.04 (m, 2H, N–CH_2_–CH_2_). ^13^C-NMR (100 MHz, DMSO) δ (ppm) = 161.6, 153.8, 146.7, 146.3, 140.3, 134.6, 133.1, 131.4, 131.3, 128.9, 128.1, 127.2, 127.0,126.3, 125.7, 120.8, 115.3, 115.0, 61.4, 52.7, 42.3, 37.0, 31.4. MS m/z (%):427.2 (0.2), 253.2 (58.8), 174.2 (100), 150.1 (6.3), 119.1 (7.9), 91.2 (19.1), 42.2 (9.8). Elem. anal. calcd. For C_27_H_26_FN_3_O (427.2); C, 75.85; H, 6.13; N, 9.83. Found: C, 75.20; H, 6.09; N, 9.81.

***2-((4-benzylpiperidin-1-yl) methyl)-3-(4-chlorophenyl) quinazolin-4(3H)-one (7f)***

IR (KBr) v (cm^−1^): 3025–3069 (C–H, aromatic), 2776–2934 (C–H, aliphatic), 1687 (C=O, Stretch), 1604 (C=C aromatic), 1492 (C=N, Stretch), 783 (C–CL, Stretch). ^1^H-NMR (500 MHz, DMSO) δ(ppm) = 8.12 (d, 1H, *J* = 7.5 Hz, Quinazoline), 7.85 (s, 1H, Quinazoline), 7.72 (d, 1H, *J* = 7.5 Hz, Quinazoline), 7.5 (s, 3H, Quinazoline and Phenyl), 7.46–7.47 (m, 2H, Phenyl), 7.24–7.25 (m, 2H, Benzyl), 7.1–7.15 (m, 3H, Benzyl), 3.18 (s, 2H, N–CH_2_–C=N), 2.42 (s, 2H, N–CH_2_), 2.35 (d, 2H, *J* = 9 Hz, N-CH_2_), 1.84 (t, 2H, *J* = 10.5 Hz, Ph–CH_2_), 1.35–1.37 (m, 3H, CH_2_–CH–CH_2_, N–CH_2_–CH_2_), 0.98 (d, 2H, *J* = 10.5 Hz, N–CH_2_–CH_2_). ^13^C-NMR (125 MHz, DMSO) δ (ppm) = 161.0, 153.0,146.2, 139.7, 135.4, 134.1, 132.5, 130.6, 128.4, 127.8, 127.5, 126.7, 126.5, 125.8, 125.1, 120.3, 60.9, 52.2, 41.7, 36.4, 30.9. MS m/z (%): 443.2 (0.1), 269.1 (47.9), 234.2 (11.8), 174.3 (100), 119.2 (13.3), 91.2 (22.07), 65.2 (3.4), 42.2 (9.4). Elem. anal. calcd. For C_27_H_26_ClN_3_O (443.9); C, 73.04; H, 5.90; N, 9.46. Found: C, 72.98; H, 5.86; N, 9.37.

***2-((4-benzylpiperidin-1-yl) methyl)-3-(4-phenoxyphenyl) quinazolin-4(3H)-one (7g)***

IR (KBr) v (cm^−1^):3025–3067 (C-H, aromatic), 2758–2907 (C-H, aliphatic), 1688 (C=O, Stretch), 1590 (C=C aromatic), 1505 (C=N, Stretch), 1163 (C-O, Stretch). ^1^H-NMR (500 MHz, DMSO) δ(ppm) = 8.23 (dd, 1H, *J* = 6.4 Hz, 1.2 Hz, Quinazoline), 7.97 (t, 1H, *J* = 7.6 Hz, Quinazoline), 7.83 (d, 1H, *J* = 7.6 Hz, Quinazoline), 7.68 (t, 1H, *J* = 7.6 Hz, Quinazoline), 7.52–7.57 (m, 4H, Phenyl), 7.16–7.37 (m, 10H, Phenyl and Benzyl), 3.31 (s, 2H, N–CH_2_–C=N), 2.46–2.53 (m, 4H, N–CH_2,_ N–CH_2_),1.99 (t, 2H, *J* = 10.8 Hz, Ph-CH_2_), 1.47–1.50 (d, 3H, *J* = 10.8, CH_2_–CH–CH_2,_ N–CH_2_–CH_2_), 1.09–1.16 (m, 2H, N–CH_2_–CH_2_). ^13^C-NMR (125 MHz, DMSO) δ(ppm) = 162.2, 157.1, 156.9, 154.4, 147.2, 140.8, 135.1, 132.6, 131.5, 130.6,129.4, 128.6, 127.7, 127.5, 126.8, 124.2, 121.3, 119.1, 118.9, 62.0, 53.3, 42.7, 37.5, 32.0. MS m/z (%):501.2 (0.4), 327.2 (13.3), 174.3 (100), 119.2 (7.4), 91.2 (13.07), 55.2 (21.6). Elem. anal. calcd. For C_33_H_31_N_3_O_2_ (501.2); C, 79.02; H, 6.23; N, 8.38. Found: C, 79.01; H, 6.19; N, 8.31.

***2-((4-benzylpiperidin-1-yl) methyl)-3-(5-chloro-2-methoxyphenyl) quinazolin-4(3H)-one (7h)***

IR (KBr) v (cm^−1^):3024–3082 (C–H, aromatic), 2807–2930 (C–H, aliphatic), 1687 (C=O, Stretch), 1608 (C=C aromatic), 1520 (C=N, Stretch), 1018 (C-O, Stretch), 699 (C–CL, Stretch). ^1^H-NMR (500 MHz, DMSO) δ(ppm) = 8.12 (s, 1H, Quinazoline),7.55–7.86 (m,5H, Quinazoline and Phenyl), 7.1–7.3 (m, 6H, Phenyl and Benzyl), 3.7–4.04 (m, 6H, aliphatic), 3.04 (s, 1H, aliphatic),1.85–2.5 (m, 5H, aliphatic), 1.35–1.46 (m, 3H, aliphatic), 0.93–0.95 (m,1H, aliphatic). ^13^C-NMR (125 MHz, DMSO) δ (ppm) = 160.3, 153.3,146.2, 144.8, 139.6, 134.4, 134.1, 130.3, 129.6, 129.2, 129.1, 128.4, 128.2, 127.5, 126.7, 126.6, 125.8, 125.1, 122.7, 120.1, 61.1, 55.7, 55.4, 44.9, 41.9, 36.1, 31.1, 30.9. MS m/z (%):473.2 (0.2), 455.2 (1.8), 269.1 (28.8), 234.2 (13.6), 174.3 (100), 154.1 (2.04), 119.2 (4.5), 91.2 (13.3), 55.2 (19.11). Elem. anal. calcd. For C_28_H_28_ClN_3_O (473.2); C, 73.43; H, 6.16; N, 9.17. Found: C, 73.35; H, 6.09; N, 9.12.

### MTT assay

Anticancer activity of all designed compounds (***7a–7h***) were achieved by standard 3-(4,5dimethylthiazol-yl)-2,5-diphenyl-tetrazolium bromide (MTT) assay according to our previous protocols [23]. Three human cancer cell lines such as MCF-7 (breast carcinoma), A549 (non-small cell lung carcinoma), 5637 (bladder carcinoma) and normal lung cell (MRC-5) were purchased from National Cell Bank of Iran (NCBI, Pasteur Institute, Tehran, Iran). All cancer cell lines were cultured in RPMI 1640 culture media. For normal cell line (MRC-5) DMEM/Ham’s F12 (Bio Idea, Iran) were used. All media were supplemented with 10% fetal bovine serum (FBS) and 1% penicillin–streptomycin (Gibco, USA). The cells were kept at 37 °C in a humidified CO_2_ incubator. The cells were harvested using trypsin/EDTA 0.5% solution (Gibco/USA) and were then seeded in 96-well microplates at a density of 1 × 10^4^ cells per well for MCF-7 cell lines and 8 × 10^3^ cells per well for A549, 5637 cell lines and 15 × 10^3^ for MRC-5 cell line in 100 μl of complete culture medium as previously determined [30]. The five different concentrations of the synthesized compounds and *cis-*platin as positive control (1 to 500 μM) were used for treatment in triplicate manner. For negative controls, three untreated wells were used. The media were replaced by 100 μL fresh MTT solution after 72 h and incubated for 4 h at 37 °C in the incubator to obtain formazan purple crystals [31]. The media were removed and 150 μL of DMSO were added and incubated at 37 °C in dark for 10 min to dissolve the crystals. The absorbance of individual well was read at 490 nm using a microplate ELISA reader. Excel 2016 and Curve Expert 1.4 were used to analyze the data. The data were presented mean ± SD for each analysis.

### In silico physicochemical parameters calculations

The SwissADME online software was used to obtain the drug-likeness and ADME properties of all compounds [32].

### Docking procedure

The PDB ID of EGFR (1M17) was obtained from Protein Data Bank (http://www.rcsb.org). All synthesized compounds were generated, optimized, and converted to pdbqt format. The final format of receptor was prepared by remove cognate ligand and water molecules and finally, missing hydrogen atoms were added and non-polar hydrogens were merged by using AutoDock Tools package (1.5.6) [33]. An in-house batch script (DOCKFACE) was applied to obtain the grid box with a size of 40 × 40 × 40 points in x, y, and z directions. The other docking parameters were set as default. Binding interactions of docked compounds and the receptor were analyzed by discovery studio client 2016 [34].

### Simulation procedure

Gaussian 09 program package was used to optimize all synthesized compounds [35]. MD simulations were performed with GPU accelerated Gromacs 2020 [36]. The topology and coordinate files for protein were obtained using pdb2gmx program taking parameters from AMBER99SB-LIDN force field. The partial atomic charges of ligands was performed based on AM1-BCC method using Antechamber program of AmberTools [37]. The full Amber topology and coordinate files of ligands were generated using parmchk and tleap programs implemented in AmberTools package [38]. Acpype python script was used to convert the AMBER format to the GROMACS format files. Afterwards, the topology file for each complex were made and also, systems were solvated in a cubic periodic box with a side length of 20 Å^3^ by addition of TIP3P water molecules, and then, they were neutralized. The prepared systems were energy-minimized with steepest descent algorithm for 50,000 steps. A NVT and NPT equilibration was done according to our previous works [39]. The final MD production run was performed in 100 ns with a 0.2 fs time step. VMD software package was applied to visualize protein–ligand complexes interactions and MD trajectories [40].

## Conclusion

In effort to find novel antiproliferative compounds, a series of quinazolinone-benzyl piperidine derivatives was prepared and identified. Their activities against three cancerous cell lines (MCF-7, A549 and 5637) were tested and their selectivity also evaluated against tumorigenic and non-tumorigenic cell lines. Most of the compounds, especially ***7b***, ***7e*** and ***7f,*** were found to have moderate activity against tested cancer cell lines, while assessment of compounds against normal cell line revealed lower toxicity. Molecular docking results also supported the cytotoxic activities of these novel compounds as EGFR inhibitors. Overall, these compounds can be used for development of new anticancer agents with further modifications.

**Table 1:** Chemical structures and physical data of the quinazoline derivatives

Entry	Chemical structures	M.W	M.P.(℃)	(%)Yield	Entry	Chemical structures	M.W	M.P.(℃)	(%)Yield
*7a*		443.98	123–127	62	***7e***		427.52	167–170	95
*7b*		.488.43	117–124	65	***7f***		443.98	158–160	57
*7c*		.469.59	119–123	57	***7g***		501.63	115–117	69
*7d*		.477.53	154–157	75	***7h***		458.0	157–160	50

**Table 2:** In vitro cytotoxic activity of the quinazoline derivatives

Compound	Cytotoxicity (IC_50_ ± SD) µM			
	MCF-7	A549	5637	MRC-5
***7a***	337.5 ± 9.6	190.2 ± 5.3	290.4 ± 7.6	> 500
***7b***	82.1 ± 5.6	67.3 ± 6.5	51.4 ± 2.5	201.9 ± 2.3
***7c***	232.8 ± 11.8	347.6 ± 25.3	139.8 ± 5.3	> 500
***7d***	266.5 ± 21.7	178.2 ± 2.9	83.5 ± 4.9	> 500
***7e***	99.7 ± 4	113.2 ± 4.56	103.0 ± 2.1	339.0 ± 2.7
***7f***	90.2 ± 7.1	227.5 ± 3.9	74.8 ± 2.4	> 500
***7g***	241.3 ± 46.3	292.3 ± 46.3	233.4 ± 39.4	> 500
***7h***	445.39 ± 42.0	175.2 ± 28.5	235.6 ± 1.0	> 500
*Cisplatin*	36.5 ± 1.9	14.8 ± 0.27	37.7 ± 1.9	45.2 ± 2.5

**Table 3:** The bonding energies (kcal/mol) and detailed interaction of the synthesized compounds toward EGFR receptors (1M17) using AutoDock Vina

Entry	Amino Acid	Ligand involved moiety	Type of interaction	B.E (kcal/mol)	Entry	Amino Acid	Ligand involved moiety	Type of interaction	B.E (kcal/mol)
***7a***	Phe 699	4-benzyl piperidine	pi-pi	− 9.6	***7e***	Thr 766	carbonyl	Hydrogen bond	− 9.6
	Val 702, Lys 851	4-benzyl piperidine, Quinazoline ring	pi-alkyl			Lys 721, Glu 738	4-F benzyl	pi-cation, halogen bond	
	Glu 734	3-Cl benzyl	Pi-anion			Phe 699, Leu 820, Val 702, Ala 719, Leu 694	4-benzyl piperidine, Quinazoline ring	pi-pi, pi-sigma	
	Arg 817, Asp 831, Cys 721, Ile 735, Glu 738, Gly 833, Leu 834	–	Vander waals			Gly 697, Gly 772, Met 769, Leu 768, Gln 767, Leu 764, Met 742, Thr 830	–	Vander waals	
***7b***	Leu 694, Leu 820, Ala 719, Val 702, Lys 721	Quinazoline ring and 3-Br benzyl	pi-alkyl	− 9.7	***7f***	Met 742, Lys 721, Leu 764, Ala 719, Leu 820,	4-Cl benzyl, Quinazoline ring	pi-alkyl	− 9.4
	Phe 699	4-benzyl piperidine	pi-pi			Val 702, Leu 694	Quinazoline ring	pi-sigma	
	Asp 831	4-benzyl piperidine	pi-anion			Arg 817	4-benzyl piperidine	pi-cation	
	Arg 817, Thr 830, Glu 738, Leu 764, Met 742, Thr 766, Met 769, Leu 768, Gly 772	–	Vander waals			Asn 819, Phe 699, Thr 830, Glu 738, Thr 766, Asp 831, Cys 773, Gly 772, Met 769, Leu 768	–	Vander waals	
***7c***	Leu 820, Val 702, Lys 721, Ala 719, Arg 817	4-benzyl piperidine, 2,4 di-methoxy benzyl	pi-alkyl	− 8.4	***7g***	Val 702	4-phenoxy benzyl	pi-sigma	− 8.9
	Phe 699	Quinazoline ring	pi-pi			Lys 721, Ala 719	4-phenoxy benzyl	Alkyl interaction	
	Asp 831	Quinazoline ring	pi-anion			Cys 773	4-benzyl piperidine	pi-alkyl	
	Gly 697, Cys 773, Thr 830, Leu 775	–	Vander waals			Glu 780, His 781, Tyr 777, Phe 771, Asp 776, Leu 694, Leu 820, Thr 830, Thr 766, Phe 699, Gly 695, Gly 697, Gly 772		Vander waals	
***7d***	Lys 721	Quinazoline ring	pi-anion	-9.9	***7h***	Arg 817	Carbon hydrogen bond		− 9.4
	Val 702, Ala 719, Leu 820, Leu 694, Leu 764, Met 742	4-benzyl piperidine, 3-CF_3_ benzyl	pi-alkyl			Ala 719, Leu 820	4-benzyl piperidine	pi-alkyl	
	Gly 695, Thr 830, Met 769, Leu 768, Gly 772, Phe 699, Asp 831, Glu 738, Thr 766	–	Vander waals			Met 769, Leu 768, Leu 694, Gly 772, Asp 776, Gly 695, Phe 699, Asp 831, Cys 773, Val 702, Thr 830	–	Vander waals

**Table 4:** Physicochemical properties of synthesized compounds

Entry	MW^a^	LogP^b^	HBD^c^	HBA^d^	TPSA (A^2^) ^e^	n-RB^f^	Lipinski violation
*7a*	443.95	4.81	0	3	38.13	5	0
*7b*	427.51	4.71	0	4	38.13	5	0
*7c*	469.57	3.37	0	5	56.59	7	0
*7d*	477.52	5.11	0	6	38.13	6	0
*7e*	427.51	4.71	0	4	38.13	5	0
*7f*	443.97	4.81	0	3	38.13	5	0
*7g*	501.62	5.4	0	4	47.36	7	0
*7h*	457.99	5.01	0	3	38.13	5	2
Rule of Lipinski	≤ 500	≤ 5	≤ 5	≤ 10	≤ 140	≤ 10	≤ 1

^a^Molecular weight (MW)

^b^Logarithm of partition coefficient between n-octanol and water (LogP)

^c^Number of hydrogen bond donors (HBD)

^d^Number of hydrogen bond acceptors (HBA)

^e^Topological polar surface area(TPSA)

^f^Number of rotatable bonds (nRB)

## Supplementary Information

**Additional file 1: Figure S1**. The FT-IR spectrum of 7a. **Figure S2**. The 1H NMR spectrum of 7a. **Figure S3**. The 13C-NMR spectrum of 7a. **Figure S4**. The Mass spectrum of 7a. **Figure S5**. The FT-IR spectrum of 7b. **Figure S6**. The 1H NMR spectrum of 7b. **Figure S7**. The 13C-NMR spectrum of 7b. **Figure S8**. The Mass spectrum of 7b. **Figure S9**. The FT-IR spectrum of 7c. **Figure S10**. The 1H NMR spectrum of 7c. **Figure S11**. The 13C-NMR spectrum of 7c. **Figure S12**. The Mass spectrum of 7c. **Figure S13**. The FT-IR spectrum of 7d. **Figure S14**. The 1H NMR spectrum of 7d. **Figure S15**. The 13C-NMR spectrum of 7d. **Figure S16**. The Mass spectrum of 7d. **Figure S17**. The FT-IR spectrum of 7e. **Figure S18**. The 1H NMR spectrum of 7e. **Figure S19**. The 13C-NMR spectrum of 7e. **Figure S20**. The Mass spectrum of 7e. **Figure S21**. The FT-IR spectrum of 7f. **Figure S22**. The 1H NMR spectrum of 7f. **Figure S23**. The 13C-NMR spectrum of 7f. **Figure S24**. The Mass spectrum of 7f. **Figure S25**. The FT-IR spectrum of 7g. **Figure S26**. The 1H NMR spectrum of 7g. **Figure S27**. The 13C-NMR spectrum of 7g. **Figure S28**. The Mass spectrum of 7g. **Figure S29**. The FT-IR spectrum of 7h. **Figure S30**. The 1H NMR spectrum of 7h. **Figure S31**. The 13C-NMR spectrum of 7h. **Figure S32**. The Mass spectrum of 7h.

## Data Availability

The data sets used and analyzed during the current study are available from the corresponding author on reasonable request. We have presented all data in the form of Tables and Figure. The PDB code (1M17) was retrieved from protein data bank (www.rcsb.org). https://www.rcsb.org/structure/1m17.

## Abbreviations

EGFR: Epidermal growth factor receptor
MDR: Multidrug resistance
ADME: Adsorption, Distribution, Metabolism and Discretion
Rg: Radius of gyration
RMSD: Root Mean Square Deviation
RMSF: Root Mean Square Fluctuation
TPSA: Total polar surface area
DIPEA: Diisopropy lethylamine
TK: Tyrosine kinase
MTT: 3-(4, 5 Dimethylthiazol-yl)-2,5-diphenyl-tetrazolium bromide
