# Supplementary material for: Synthesis, biological evaluation, and computational studies of some novel quinazoline derivatives as anticancer agents
Source: BMC Chem. 2022 Nov 22;16(1):100. doi: 10.1186/s13065-022-00893-z (PMC9682696; doi:10.1186/s13065-022-00893-z)
Supplement: Supplementary file 1 — Additional file 1: Figure S1. The FT-IR spectrum of 7a. Figure S2. The 1H NMR spectrum of 7a. Figure S3. The 13C-NMR spectrum of 7a. Figure S4. The Mass spectrum of 7a. Figure S5. The FT-IR spectrum of 7b. Figure S6. The 1H NMR spectrum of 7b. Figure S7. The 13C-NMR spectrum of 7b. Figure S8. The Mass spectrum of 7b. Figure S9. The FT-IR spectrum of 7c. Figure S10. The 1H NMR spectrum of 7c. Figure S11. The 13C-NMR spectrum of 7c. Figure S12. The Mass spectrum of 7c. Figure S13. The FT-IR spectrum of 7d. Figure S14. The 1H NMR spectrum of 7d. Figure S15. The 13C-NMR spectrum of 7d. Figure S16. The Mass spectrum of 7d. Figure S17. The FT-IR spectrum of 7e. Figure S18. The 1H NMR spectrum of 7e. Figure S19. The 13C-NMR spectrum of 7e. Figure S20. The Mass spectrum of 7e. Figure S21. The FT-IR spectrum of 7f. Figure S22. The 1H NMR spectrum of 7f. Figure S23. The 13C-NMR spectrum of 7f. Figure S24. The Mass spectrum of 7f. Figure S25. The FT-IR spectrum of 7g. Figure S26. The 1H NMR spectrum of 7g. Figure S27. The 13C-NMR spectrum of 7g. Figure S28. The Mass spectrum of 7g. Figure S29. The FT-IR spectrum of 7h. Figure S30. The 1H NMR spectrum of 7h. Figure S31. The 13C-NMR spectrum of 7h. Figure S32. The Mass spectrum of 7h. [file 13065_2022_893_MOESM1_ESM.docx]

**Synthesis, Biological Evaluation, and Computational Studies of Some Novel Quinazoline Derivatives as Anticancer Agents**

Leila Emami^1^, Soghra Khabnadideh^1,2^, Zahra Faghih^3^, Farnoosh Farahvasi^1,2^, Fatemeh Zonobi^1,2^, Saman Zare Gheshlaghi^4^, Shadi Daili^5^, Ali Ebrahimi^4^, Zeinab Faghih^1^^[[1]](#footnote-1)^*

*^1^Pharmaceutical Sciences Research Center, Shiraz University of Medical Sciences, Shiraz, I.R.Iran.*

*^2^Faculty of Pharmacy, Shiraz University of Medical Sciences, Shiraz, I.R.Iran.*

*^3^* *Shiraz Institute for Cancer Research, Medical School, Shiraz University of Medical Sciences, Shiraz, Iran*

*^4^Department of Chemistry, Computational Quantum Chemistry Laboratory, University of Sistan and Baluchestan*

*^5^ Department of Physical and Environmental Sciences, University of Toronto Scarborough, 1265 Military Trail, Toronto, Ontario M1C1A4, Canada*


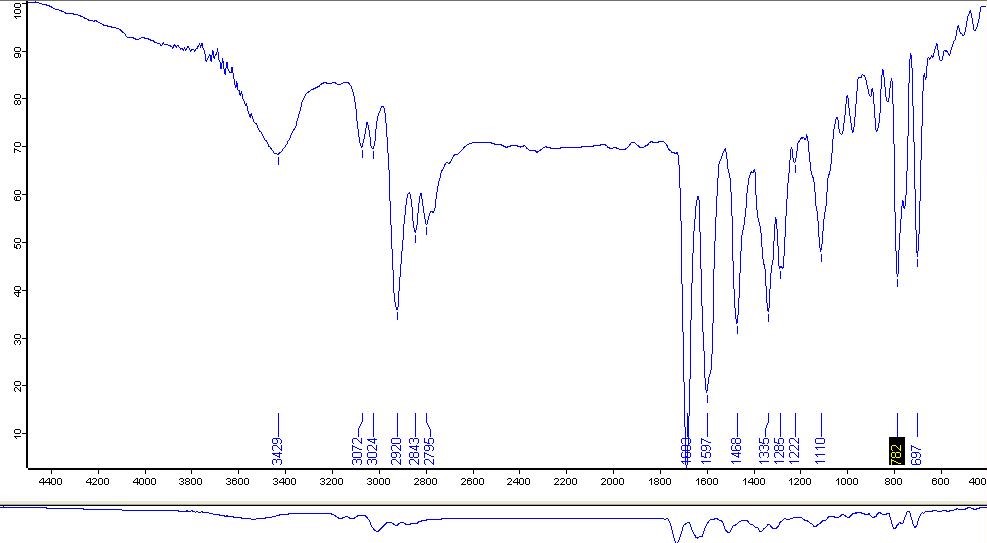


**Figure S1**. The FT-IR spectrum of ***7a***


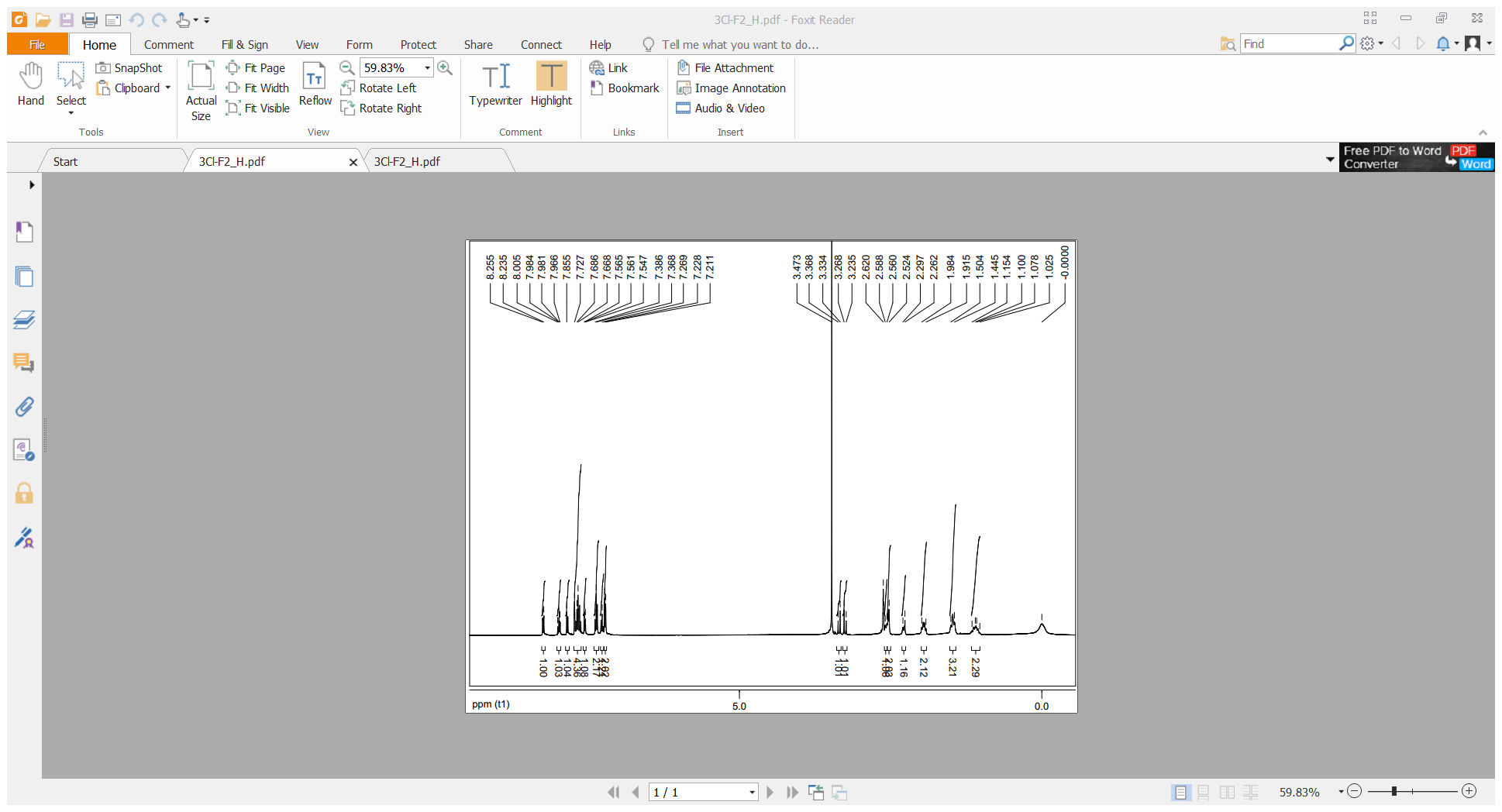


**Figure S2**. The ^1^H NMR spectrum of ***7a***


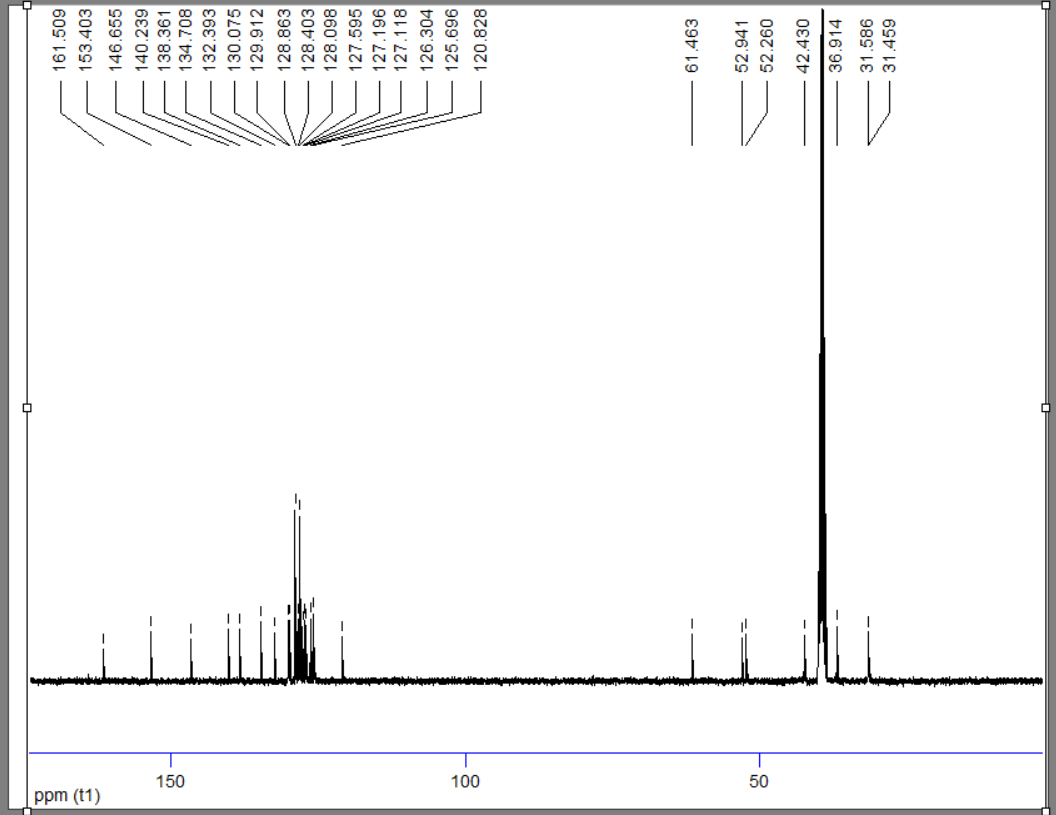


**Figure S3**. The ^13^C-NMR spectrum of ***7a***


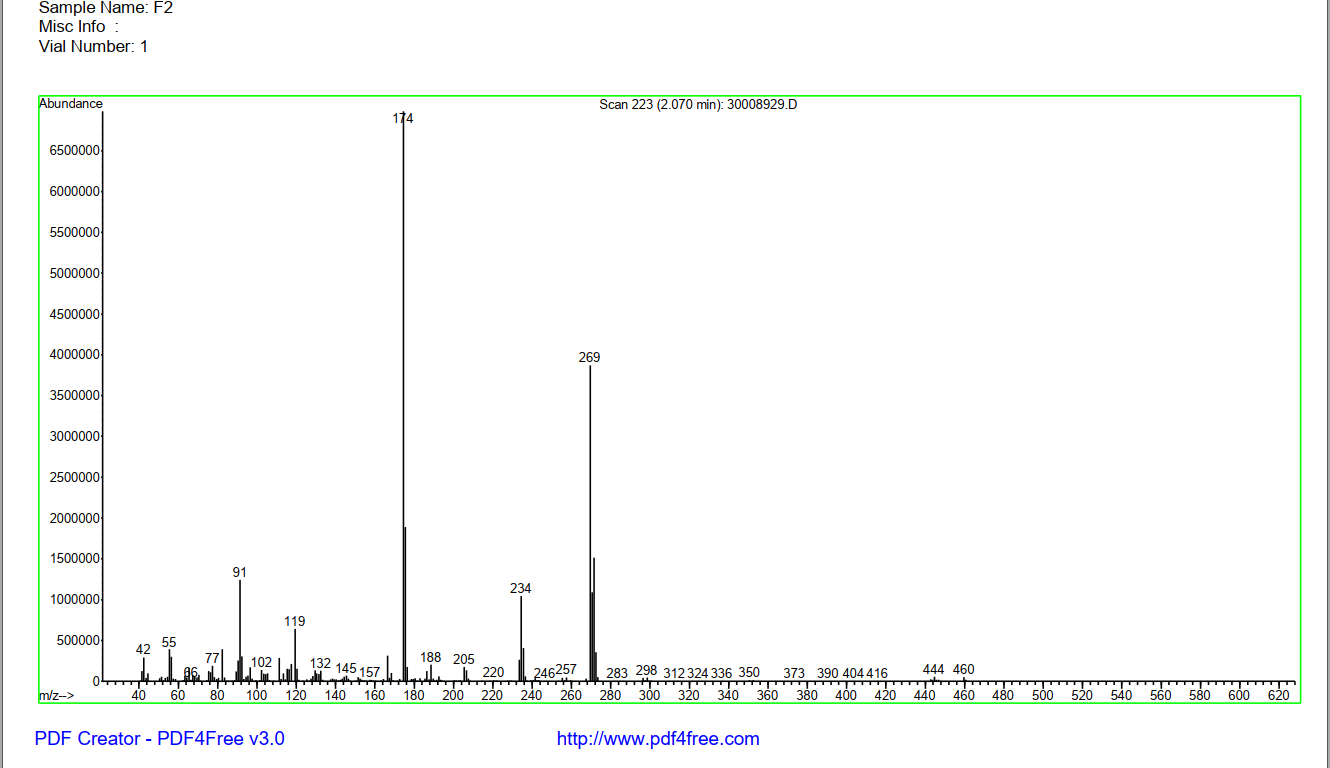


**Figure S4**. The Mass spectrum of ***7a***


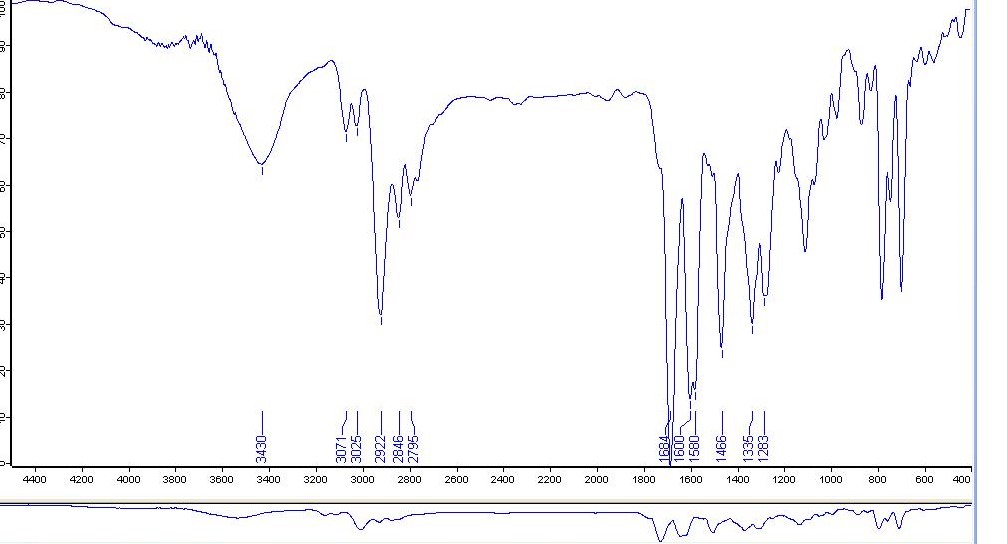


**Figure S5**. The FT-IR spectrum of ***7b***


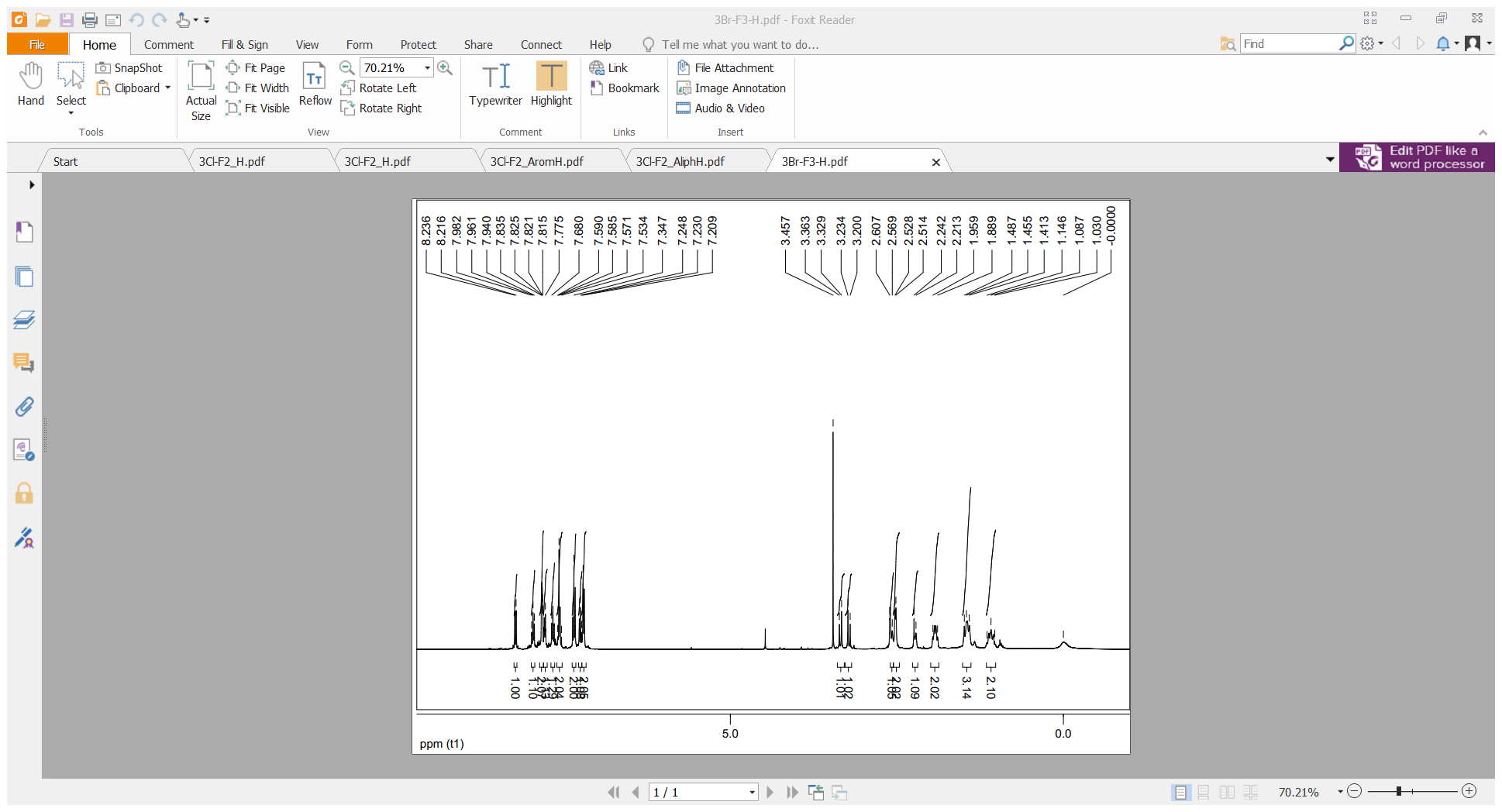


**Figure S6**. The ^1^H NMR spectrum of ***7b***


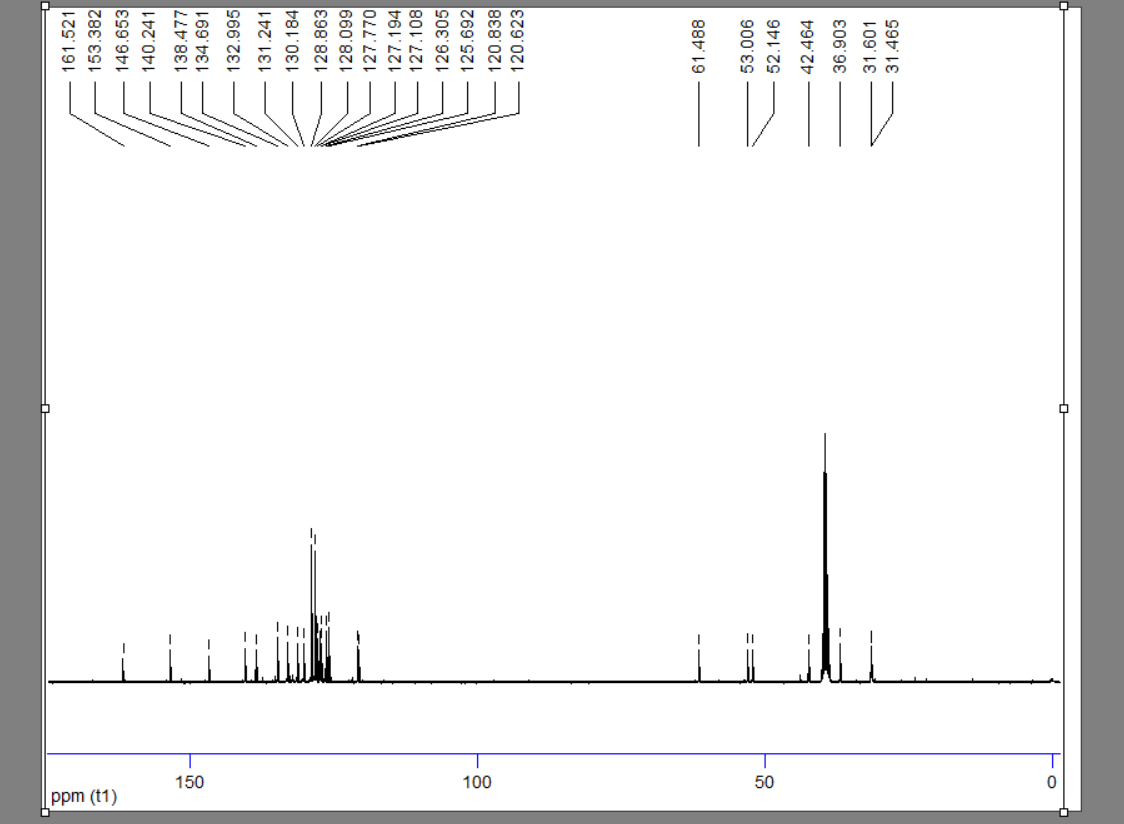


**Figure S7**. The ^13^C-NMR spectrum of ***7b***


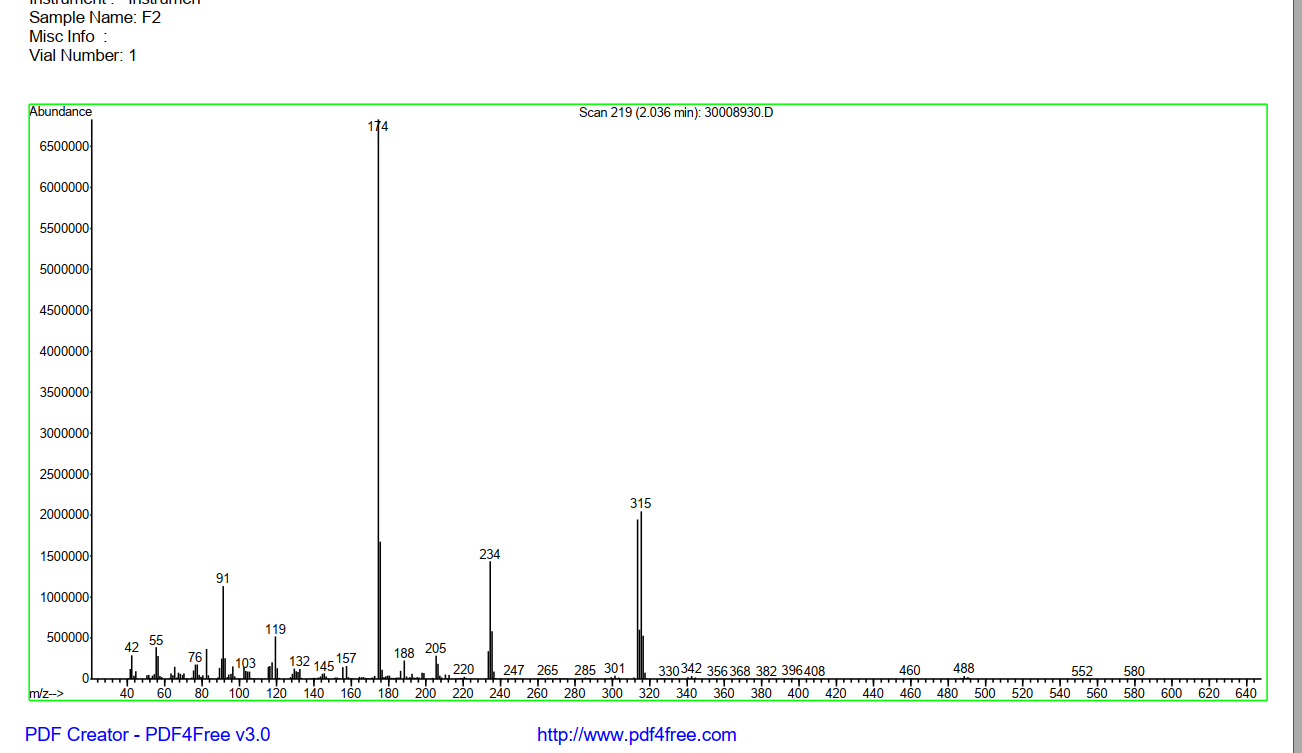


**Figure S8**. The Mass spectrum of ***7b***


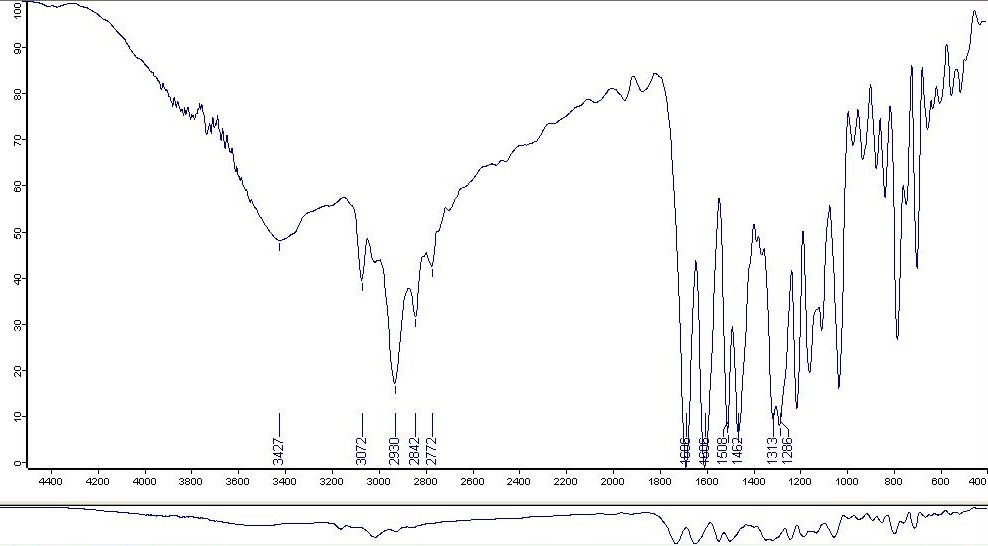


**Figure S9**. The FT-IR spectrum of ***7c***


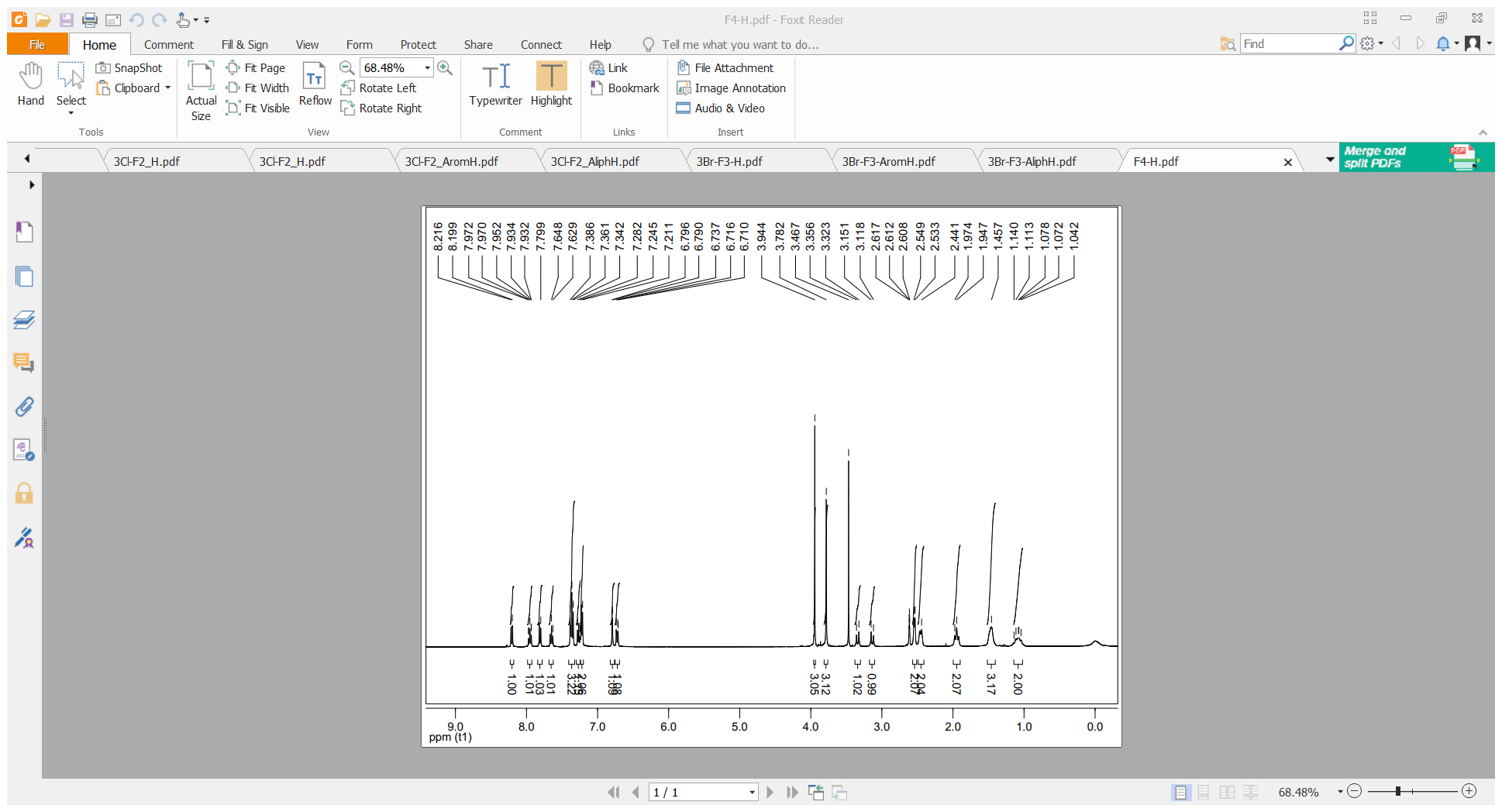


**Figure S10**. The ^1^H NMR spectrum of ***7c***


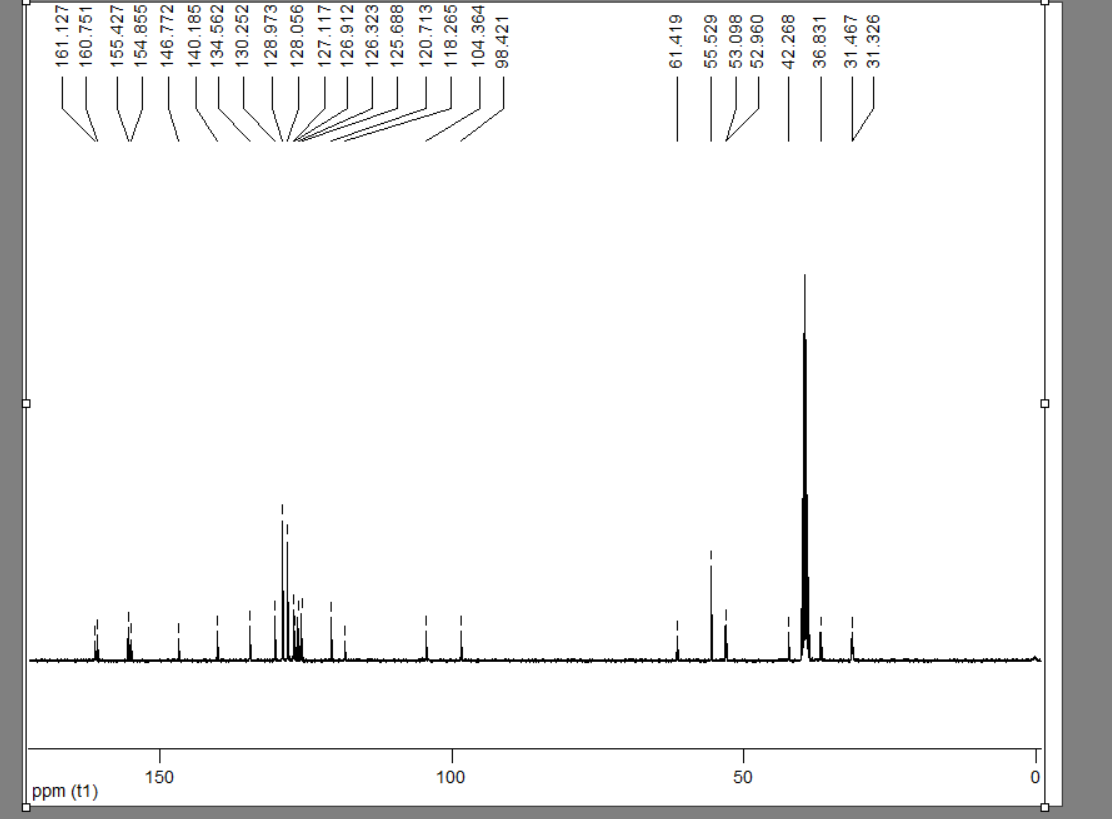


**Figure S11**. The ^13^C-NMR spectrum of ***7c***


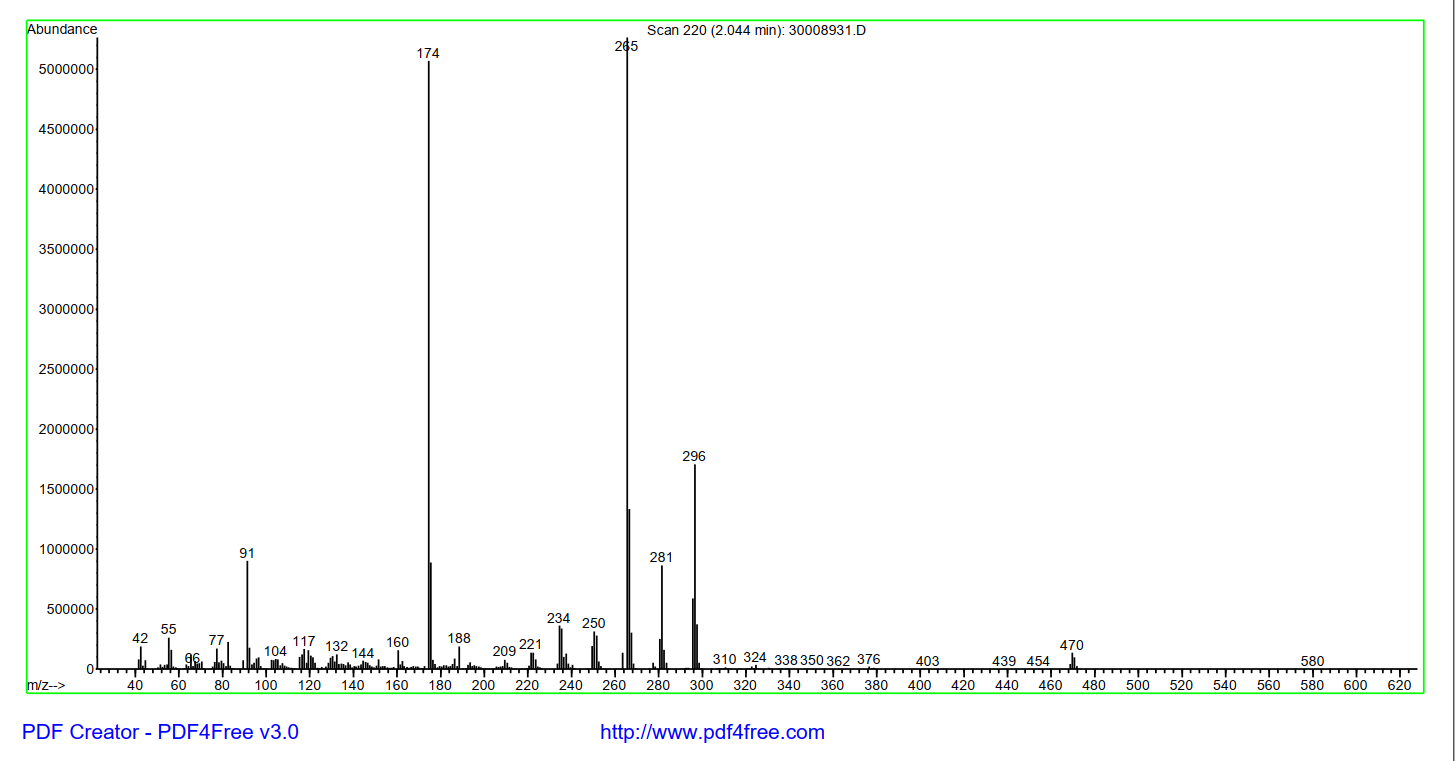


**Figure S12**. The Mass spectrum of ***7c***


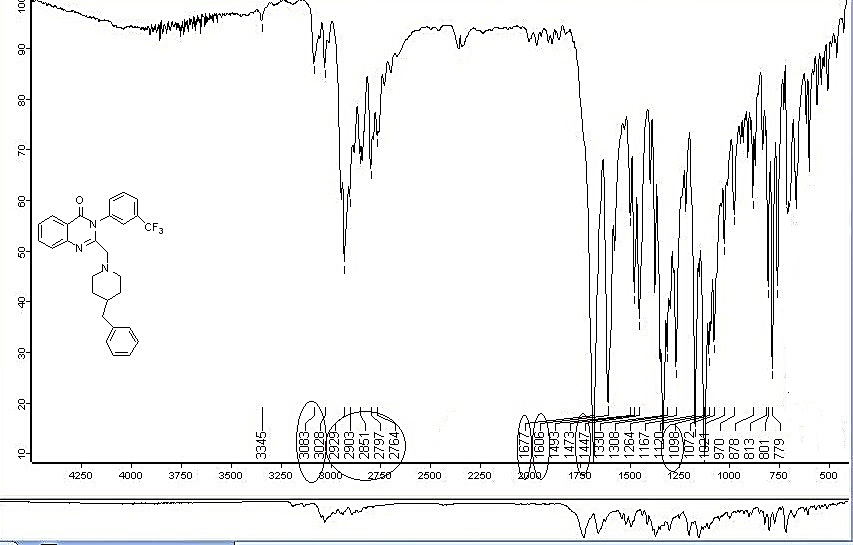


**Figure S13**. The FT-IR spectrum of ***7d***


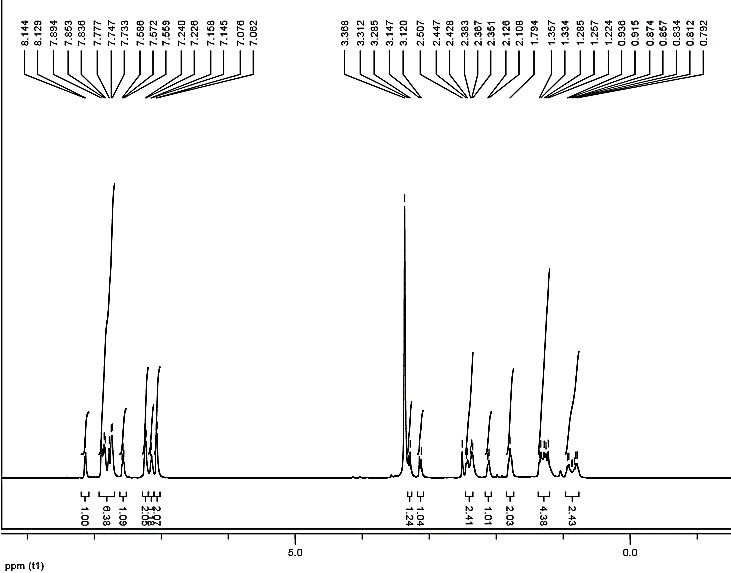


**Figure S14**. The ^1^H NMR spectrum of ***7d***


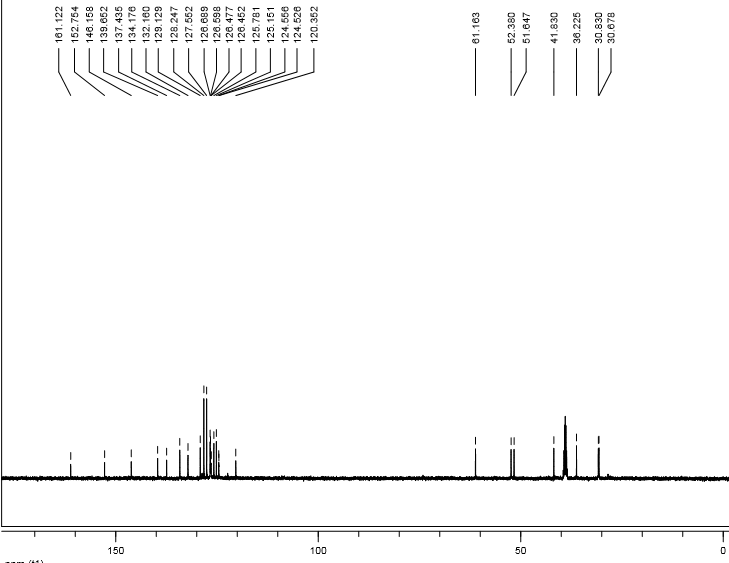


**Figure S15**. The ^13^C-NMR spectrum of ***7d***


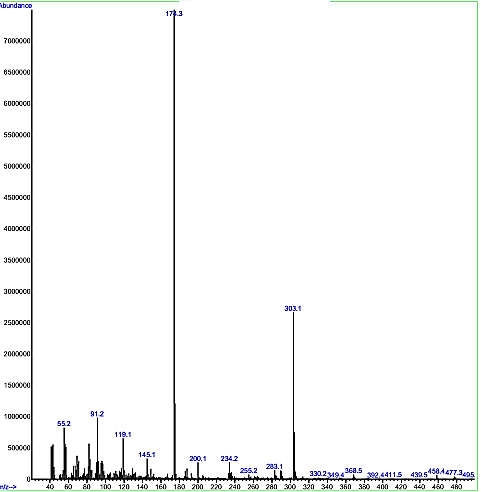


**Figure S16**. The Mass spectrum of ***7d***


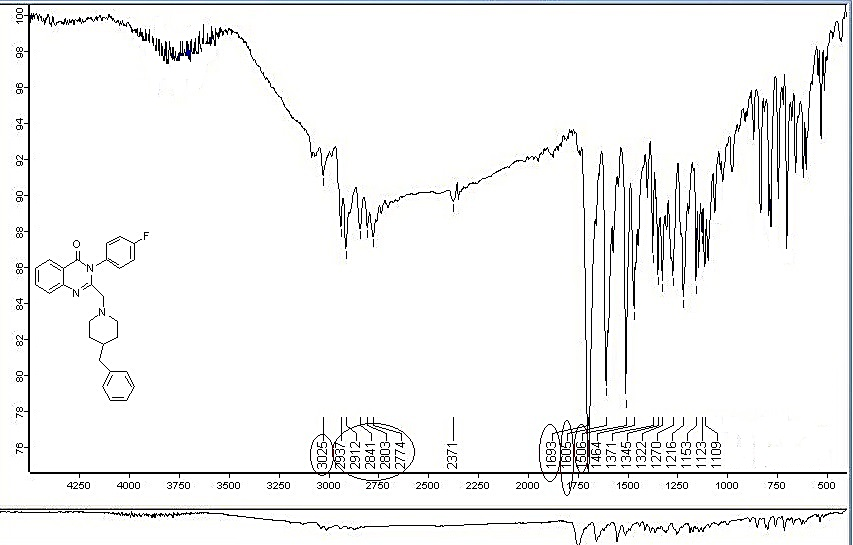


**Figure S17**. The FT-IR spectrum of ***7e***


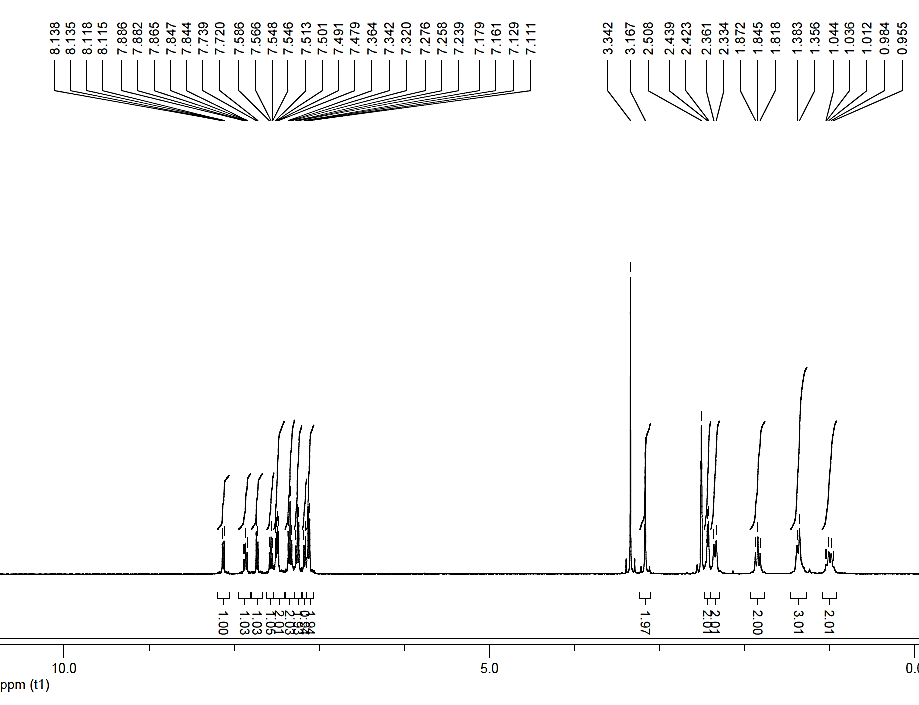


**Figure S18**. The ^1^H NMR spectrum of ***7e***


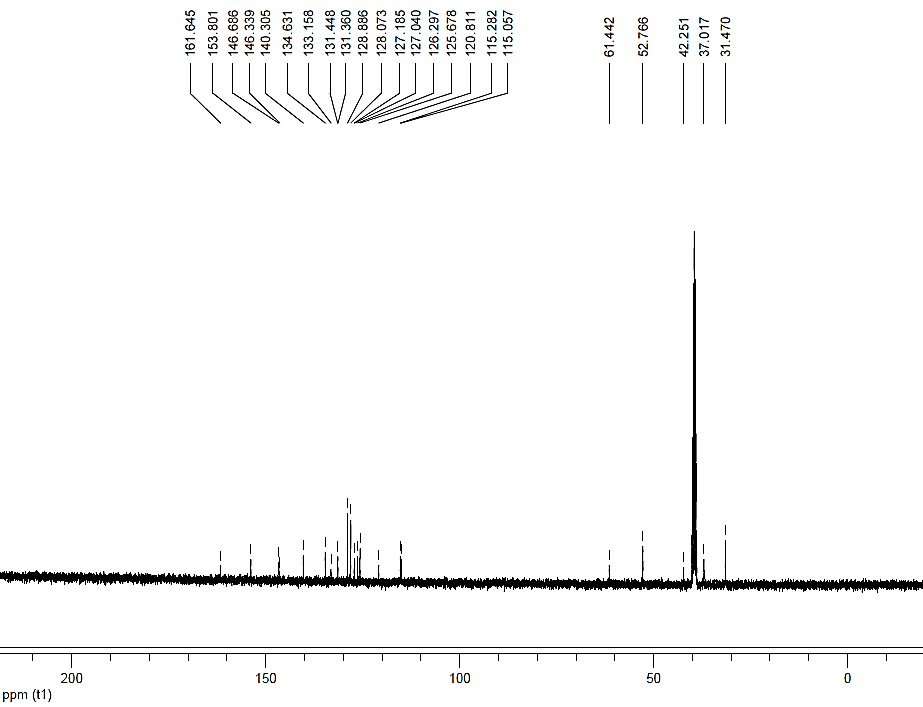


**Figure S19**. The ^13^C-NMR spectrum of ***7e***


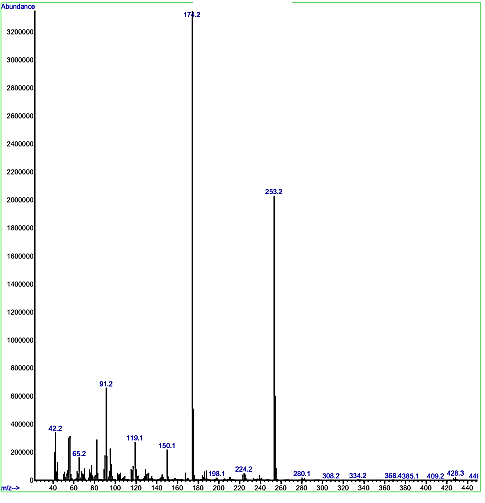


**Figure S20**. The Mass spectrum of ***7e***


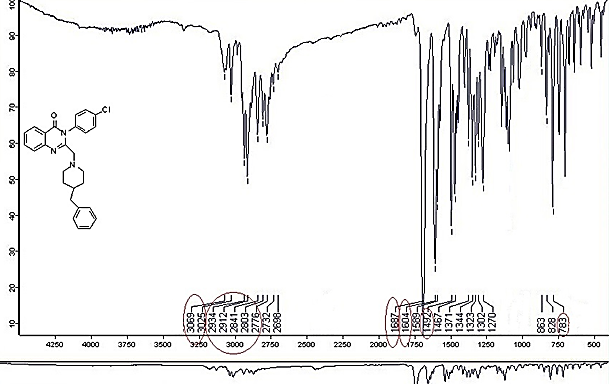


**Figure S21**. The FT-IR spectrum of ***7f***


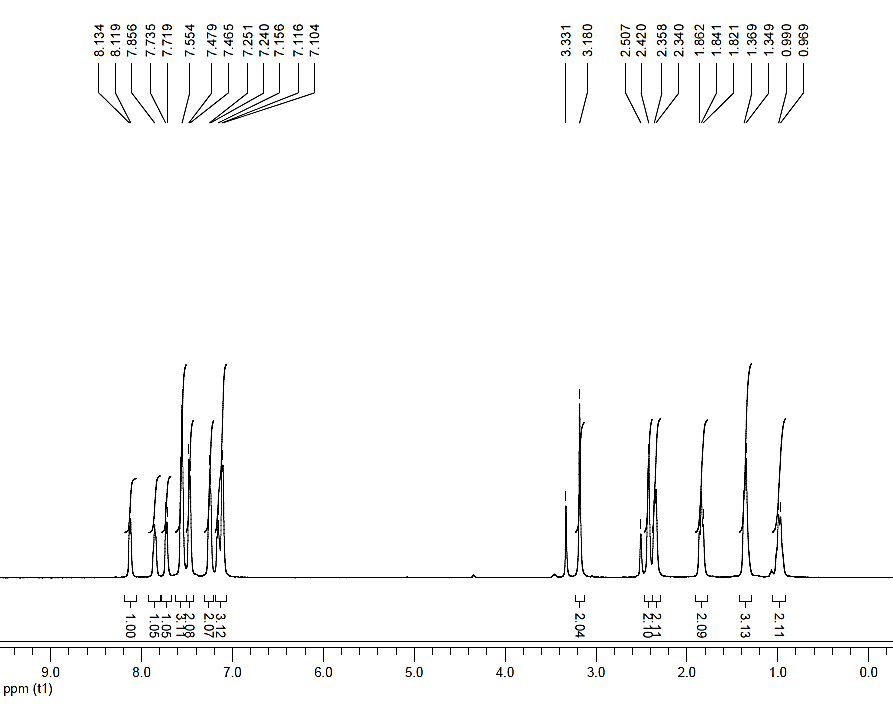


**Figure S22**. The ^1^H NMR spectrum of ***7f***


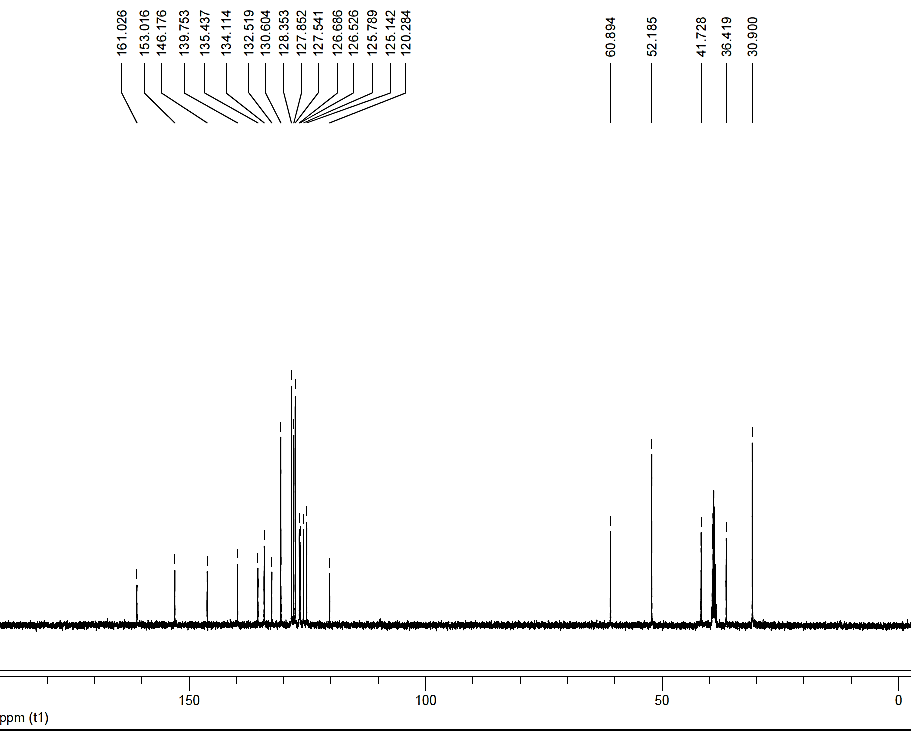


**Figure S23**. The ^13^C-NMR spectrum of ***7f***


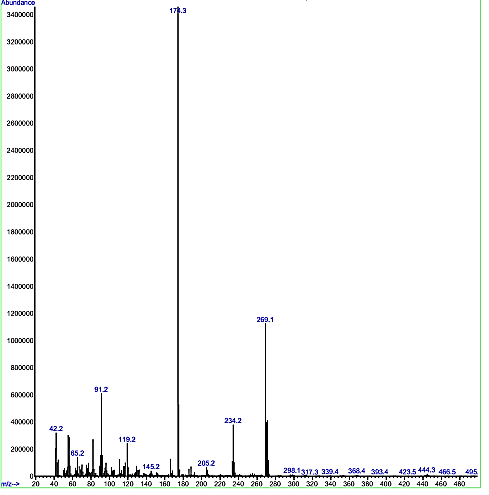


**Figure S24**. The Mass spectrum of ***7f***


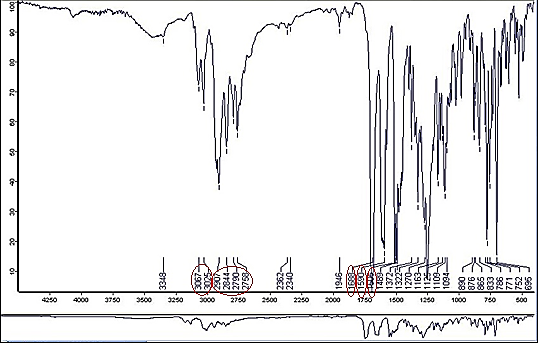


**Figure S25**. The FT-IR spectrum of ***7g***


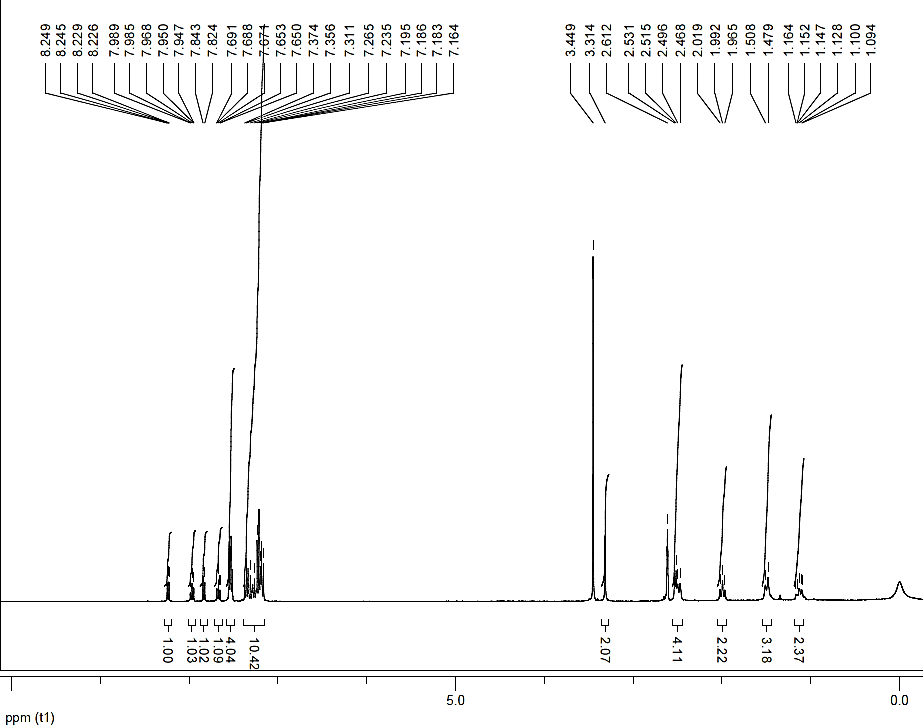


**Figure S26**. The ^1^H NMR spectrum of ***7g***


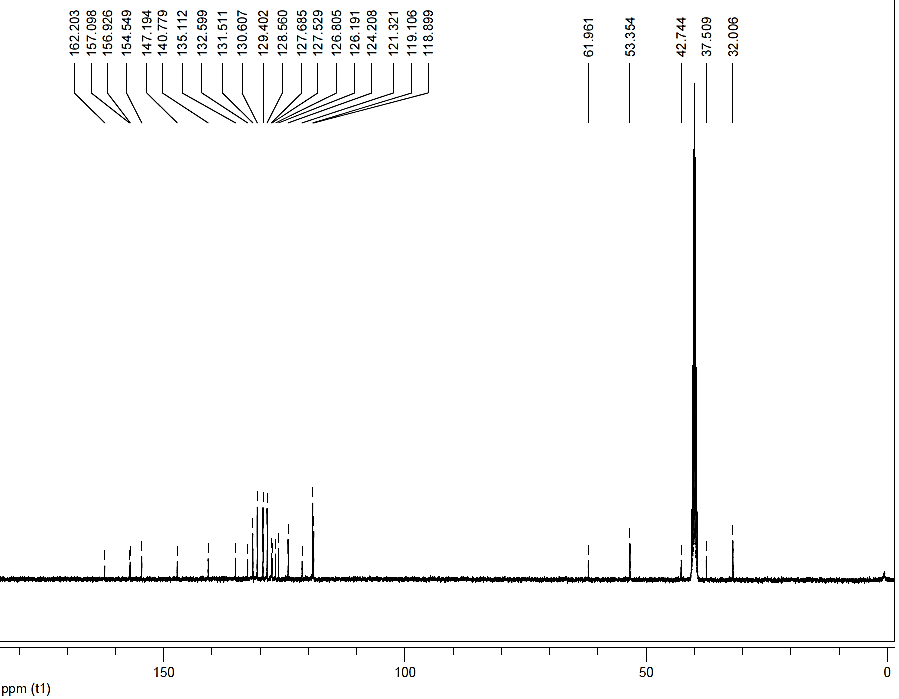


**Figure S27**. The ^13^C-NMR spectrum of ***7g***


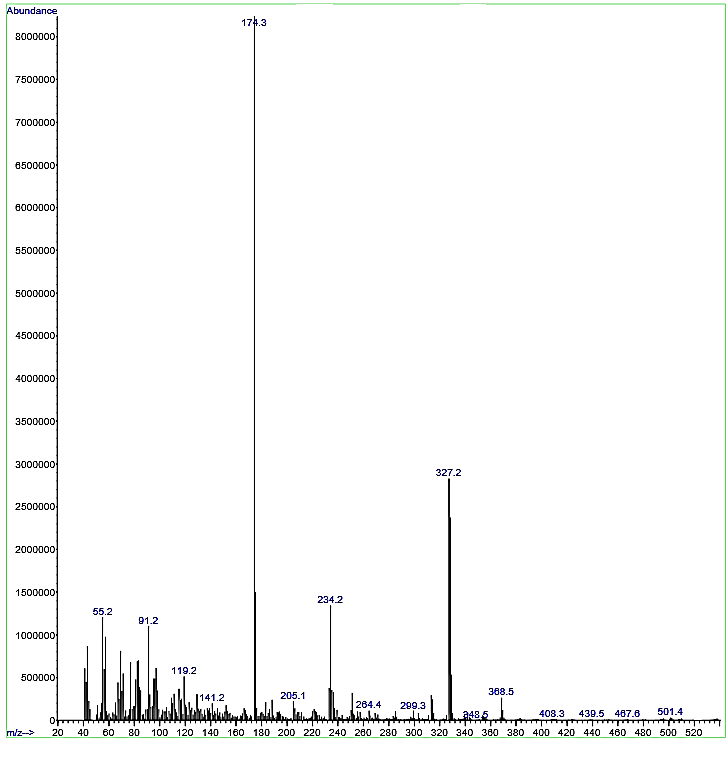


**Figure S28**. The Mass spectrum of ***7g***


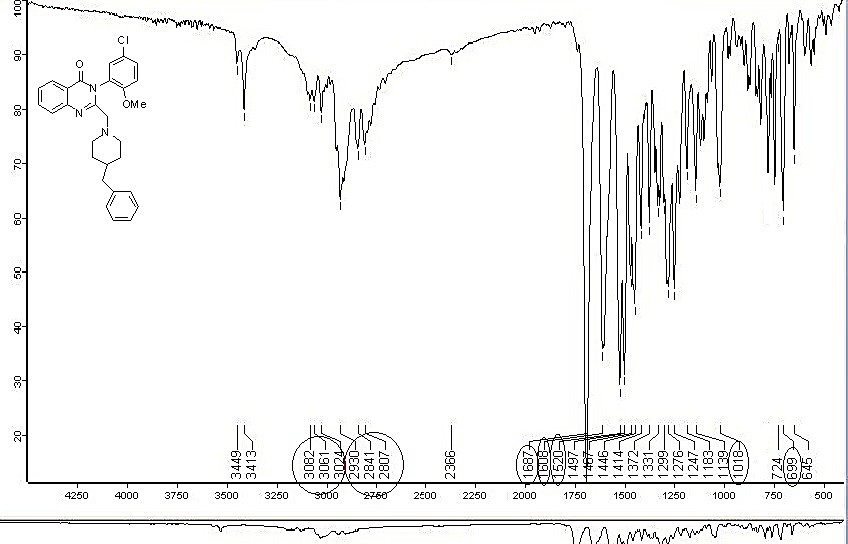


**Figure S29**. The FT-IR spectrum of ***7h***


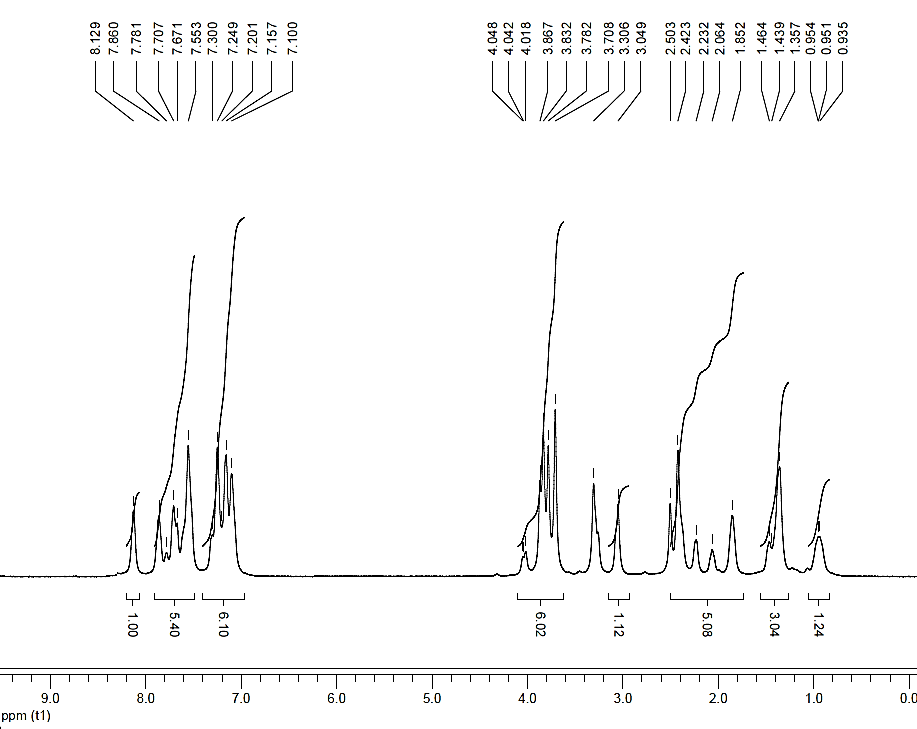


**Figure S30**. The ^1^H NMR spectrum of ***7h***


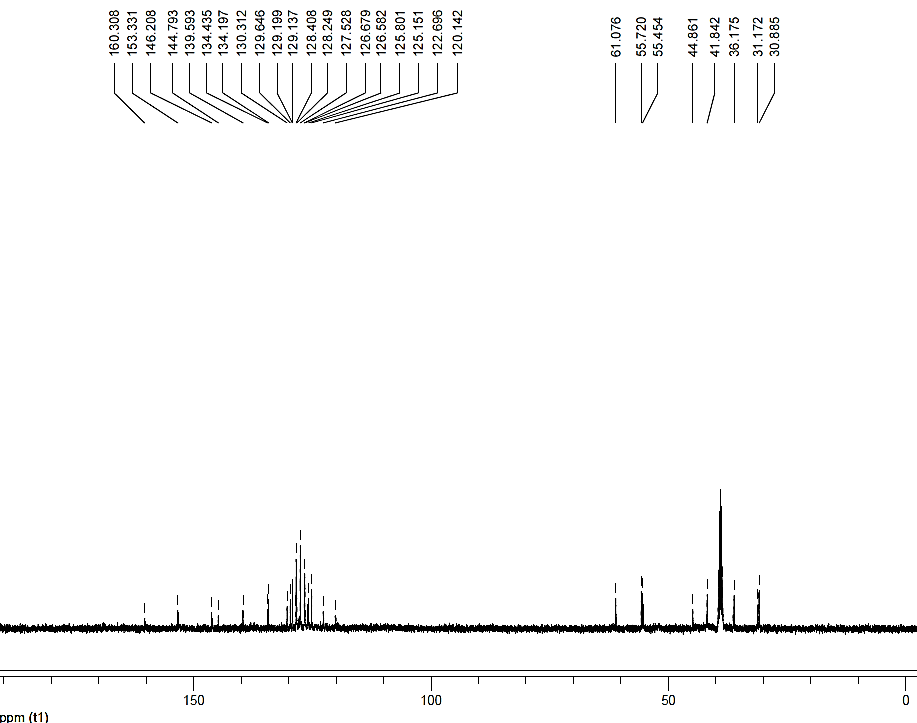


**Figure S31**. The ^13^C-NMR spectrum of ***7h***


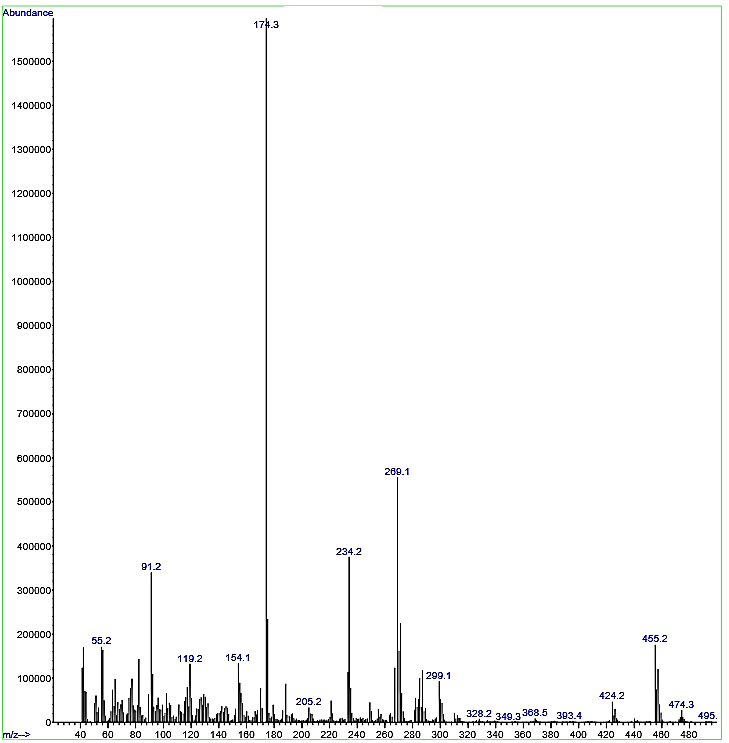


**Figure S32**. The Mass spectrum of ***7h***

1. Corresponde author:

   Zeinab Faghih, Pharmaceutical Sciences Research Center, Shiraz University of Medical Sciences, P.O. Box: 71345-1798 Shiraz, Iran. Email: [layafaghih@gmail.com](mailto:layafaghih@gmail.com) [↑](#footnote-ref-1)
